# Oncogenic PIK3CA reprograms glutamine metabolism to drive bladder cancer progression

**DOI:** 10.1186/s12964-026-03058-w

**Published:** 2026-07-14

**Authors:** Karthik Reddy Kami Reddy, Vasanta Putluri, Danthasinghe Waduge Badrajee Piyarathna, Jun Hyoung Park, Aaditya Krishna Arun, Sachin B Jorvekar, Yongchao Dou, Mohammed Khurshidul Hassan, Chandra Sekhar Amara, Abu Hena Mostafa Kamal, Chandra Shekar R. Ambati, Jun Xu, Daniel Kraushaar, Adriana Langer Gramer, Allison C Bellman, Leomar Y Ballester, Yuan Xu, Robert Taylor Ripley, Seth P Lerner, Roni J Bollag, Martha K Terris, Chong-Xian Pan, Benny Abraham Kaipparettu, Yair Lotan, Nagireddy Putluri

**Affiliations:** 1 Department of Molecular and Cellular Biology, Baylor College of Medicine, One Baylor Plaza, Houston, Texas USA; 2 Dan L Duncan Comprehensive Cancer Center, Baylor College of Medicine, Houston, Texas USA; 3 Advanced Technology Cores, Baylor College of Medicine, Houston, Texas USA; 4 Department of Molecular and Human Genetics, Baylor College of Medicine, Houston, Texas USA; 5 Georgia Cancer Center, Augusta University, Augusta, Georgia USA; 6 Division of Pathology and Laboratory Medicine, The University of Texas MD Anderson Cancer Center, Houston, Texas USA; 7 Division of Thoracic Surgery, Michael E. DeBakey Department of Surgery, Baylor College of Medicine, Houston, Texas USA; 8 Scott Department of Urology, Baylor College of Medicine, Houston, Texas USA; 9 Department of Urology, Medical College of Georgia, Augusta, Georgia USA; 10 Department of Medicine, Harvard Medical School, Veterans Affairs Boston Healthcare System, Boston, Massachusetts USA; 11 Department of Urology, University of Texas Southwestern Medical Center, Dallas, Texas USA

## Abstract

**Background:**

Genomic analysis has revealed that approximately 40% of bladder cancer (BLCA) tumors harbor alterations in the PI3K/AKT pathway, with PIK3CA mutations occurring in 15–25% of cases. PIK3CA, which encodes the catalytic p110α subunit of PI3K, plays a critical role in regulating cell survival, proliferation, and metabolism. However, the metabolic and functional consequences of PIK3CA mutations in BLCA remain poorly defined.

**Methods:**

To investigate the role of PIK3CA mutations in BLCA, we performed targeted sequencing on tumors from patients, identifying recurrent alterations. Using CRISPR/Cas9 knock-in models in SCaBER and UM-UC-3 cell lines, we introduced the PIK3CA E545K mutation to study its effects. We conducted transcriptomic profiling, targeted metabolomics, and stable isotope tracing to assess metabolic reprogramming. Functional assays measured proliferation, mitochondrial complex I activity, and glutaminolysis. Orthotopic xenografts in mice were used to evaluate in vivo tumor growth and metabolism.

**Results:**

PIK3CA mutations were present in 20% of cases, consistent with TCGA data. The E545K and E545Q hotspots accounted for 70% of these mutations. PIK3CA E545K strongly activated PI3K/AKT signaling. Transcriptomic analysis revealed enrichment of OXPHOS, fatty acid metabolism, and mTORC1 signaling. Metabolomics indicated changes in TCA cycle metabolites and enhanced reductive carboxylation of glutamine to citrate, driving fatty acid synthesis. Mutant cells showed increased expression of GLS1 and FASN, higher proliferation rates, and elevated mitochondrial complex I activity. In vivo, PIK3CA-mutant xenografts displayed significantly increased tumor growth.

**Conclusion:**

PIK3CA mutations are frequent drivers of metabolic reprogramming in BLCA, leading to increased glutamine flux, elevated OXPHOS activity, and enhanced fatty acid synthesis, all of which contribute to tumor progression. These findings provide the first comprehensive evidence that PIK3CA-driven metabolic alterations are both biomarkers of aggressive disease and actionable therapeutic targets. The efficacy of PI3Kα inhibition in combination with metabolic targets may support its potential in precision medicine for PIK3CA-mutant BLCA and highlights the value of integrating metabolic biomarkers into treatment strategies for advanced BLCA.

**Supplementary Information:**

The online version contains supplementary material available at https://doi.org/10.1186/s12964-026-03058-w.

## Introduction

Bladder cancer (BLCA) is an aggressive malignancy and one of the major cause of cancer related deaths globally [1]. BLCA is among the most prevalent cancers: the International Agency for Research on Cancer (IARC) reports that in 2022, over 600,000 individuals were newly diagnosed with BLCA, and it ranked ninth among the most commonly diagnosed cancers worldwide [2–4]. In the United States, the American Cancer Society projects approximately 84,530 new BLCA cases in 2026 accounting for about ~ 4% of all new cancer diagnoses of which over 64,730 are expected to occur in men, reflecting a substantial male predominance [5]. Clinically, BLCA poses significant challenges: nearly 6% of patients present with metastatic disease at diagnosis, and post-radical cystectomy recurrence both local and distant affects a substantial proportion of patients (up to 50%) [6, 7]. Despite improvements in diagnostics and care, treatment options for advanced BLCA are expanding.Enfortumab Vedotin and Pembrolizumab are the first-line therapy for advanced urothelial cancer [8, 9]. Upon disease progression, limited treatment options are available and only one targeted therapy has gained FDA approval.

At the genomic level, BLCA is characterized by one of the highest mutational burdens among solid tumors, with frequent alterations in genes such as TP53, TSC1, KDM6A, RB1, and PIK3CA [10]. Approximately 70% of advanced urothelial carcinomas harbor activating mutations in PIK3CA and AKT1, or inactivating mutations in PTEN, PIK3R1, and TSC1/2 [11]. PI3K is comprised of an 85 kDa regulatory subunit and a 110 kDa catalytic subunit. Increased PI3Kα signaling may occur by various mechanisms, including somatic mutations and amplification of genes involved in the PI3Kα pathway. The p110α subunit consists of five domains: the adaptor-binding domain (ABD), the RAS-binding domain (RBD), the C2 helical domain, and kinase domains [12]. Somatic mutations in p110α are frequently observed in a several human tumor types, including breast, colon, endometrial cancers, glioblastomas and BLCA [13, 14]. The PIK3CA gene encodes the catalytic p110α subunit of PI3K, and the hotspot mutations E542K, E545K/Q, and H1047R are recurrently observed in BLCA and other malignancies [15]. Urothelial cancer specific mouse models have further confirmed that PIK3CA mutations can initiate dysplasia and cooperate with other oncogenic alterations to drive bladder tumorigenesis [16].

Beyond its canonical role in regulating proliferation, survival, and apoptosis, the PI3K/AKT pathway drives profound metabolic reprogramming in cancer cells. Activating PIK3CA mutations remodel tumor metabolism by enhancing glycolysis, oxidative phosphorylation (OXPHOS), glutamine anaplerosis, reductive carboxylation, and lipid biosynthesis [17–19]. For example, cancer cells with PIK3CA mutations demonstrate dependency on α-ketoglutarate dehydrogenase, such that its inhibition disrupts the malate–aspartate shuttle and impairs proliferation [20]. Similarly, PIK3CA mutations promote metabolic pathways and tumor growth via the β-catenin/SIRT3 axis [21]. In colorectal cancer, PIK3CA mutations increase glutamine flux into the TCA cycle, fueling α-ketoglutarate production and ATP generation [22]. Aberrant PIK3CA expression is also associated with glycolytic and cholesterol biosynthetic gene signatures in BLCA [23]. Moreover, PIK3CA-driven cancers exhibit distinct metabolic fingerprints, including accumulation of arachidonic acid and eicosanoids that stimulate proliferation [24]. Additionally, glutathione metabolism represents a metabolic vulnerability, where inhibition reduces tumor cell growth [25].

Together, these findings highlight PIK3CA mutations as both oncogenic drivers and critical regulators of metabolic rewiring in BLCA. The present prospective study was therefore designed to comprehensively profile PIK3CA and other recurrent mutations in BLCA patients, and to assess their impact on tumor metabolism. By delineating these genetic–metabolic interactions, our goal is to identify novel therapeutic vulnerabilities and provide a rationale for biomarker-guided targeted therapy in advanced BLCA.

## Results

### Targeted mutation profiling of BLCA patients

**Fig. 1:**
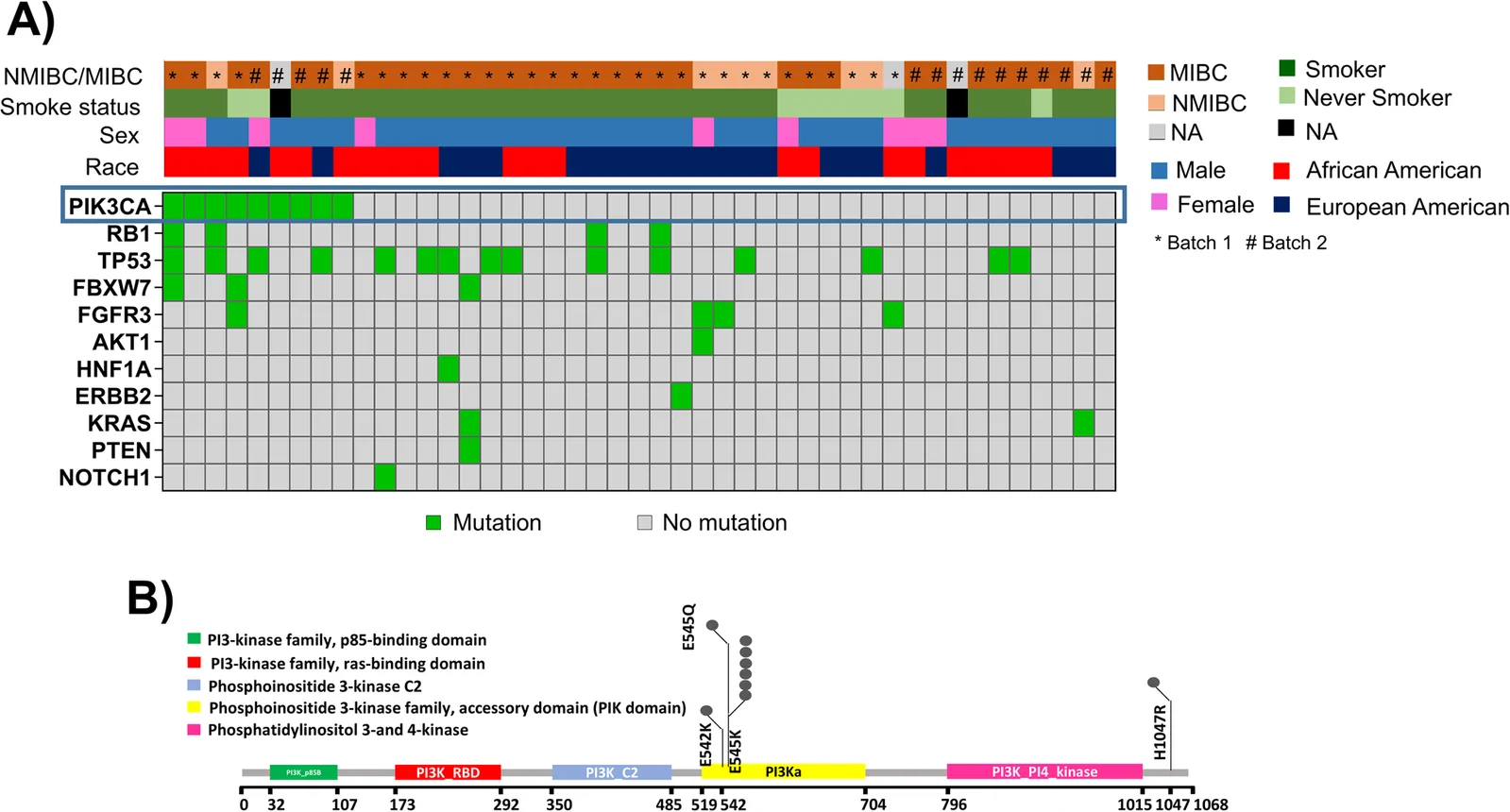
Targeted mutation analysis of BLCA patients. **A** Oncoprint showing the most frequently mutated genes in BLCA patients (n=45) identified by targeted mutation analysis [Note: Green represent mutations and grey represent no mutations]. PIK3CA mutations were detected in 20% of BLCA patients. (Note: Ta/T1 considered as NMIBC; T2 or >T2 considered as MIBC; * represent sample belongs to batch1 and # represent sample belongs to batch2; patient’s samples are arranged based on the PIK3CA mutations. Columns indicate patients and rows indicate genes; NA represents not available). **B** Lollipop plot illustrates the number of patients with PIK3CA somatic mutations observed. Grey circles represent mutation sites. Eight patients (out of 45) had PIK3CA mutations located near the α-domain (n = 1 E542K; n = 6 E545K; n = 1 E545Q). One patient harbored PIK3CA mutations in the kinase domain (n = 1 with H1047R). Note: PI3K_p85b indicates: Region of p110 PI3K that binds the p85 subunit; PI3K_rbd indicates: Phosphatidylinositol 3-kinase Ras-binding domain; PI3K_C2 indicates: Phosphatidylinositol 3-kinase, region postulated to contain C2 domain; PI3Ka indicates: Phosphoinositide 3-kinase family, accessory domain (PIK domain); PI3K_PI4_kinase indicates: Catalytic kinase domain of PI3K

In this current study, BLCA patients’ tissue samples were included for the mutation analysis. All the clinical parameters are provided in Supplementary Table 01. To identify the mutational landscape and frequency of the cancer genes, we examined genetic alterations using (PCR)-based library preparation, Illumina sequencing by synthesis (SBS) next-generation sequencing (NGS) technology, and automated analysis. This PCR-based sequencing assay was designed to detect genetic alterations (single nucleotide changes and small insertion and deletions) in selected hotspot mutation probes targeting 50 known cancer associated genes with ~ 2800 Catalogue of Somatic Mutations in Cancer (COSMIC) mutations (Cat # 20019161, Illumina, Inc). Our analysis identified mutations in known cancer associated genes in BLCA patients (Fig. 1A**).** Among these nearly ~ 33% of patients had TP53 (R280T, E258Q, L111M, R306*, E285K, P278S, E271K, P278L, P151A, L252Sfs*93, E271K, R248W, D281H), 20% of patients had PIK3CA (E542K, E545Q/K, H1047R), ~ 8% patients had FGFR3 (Y375C, S249C, R248C) and ~ 8% patients had RB1 (S576*, S149*, R358*, 463*) mutations (Refer to Supporting data 1). Concurrently with this data, we observed that other BLCA/urothelial carcinoma cohorts have ~ 12–25% PIK3CA mutations [13] (Supplementary Fig. 1A). Of note, E545K mutation was the most predominant mutation (~ 66%) (Fig. 1B).

### Generation of endogenous PIK3CA^*E545K*^ expression in human BLCA

**Fig. 2:**
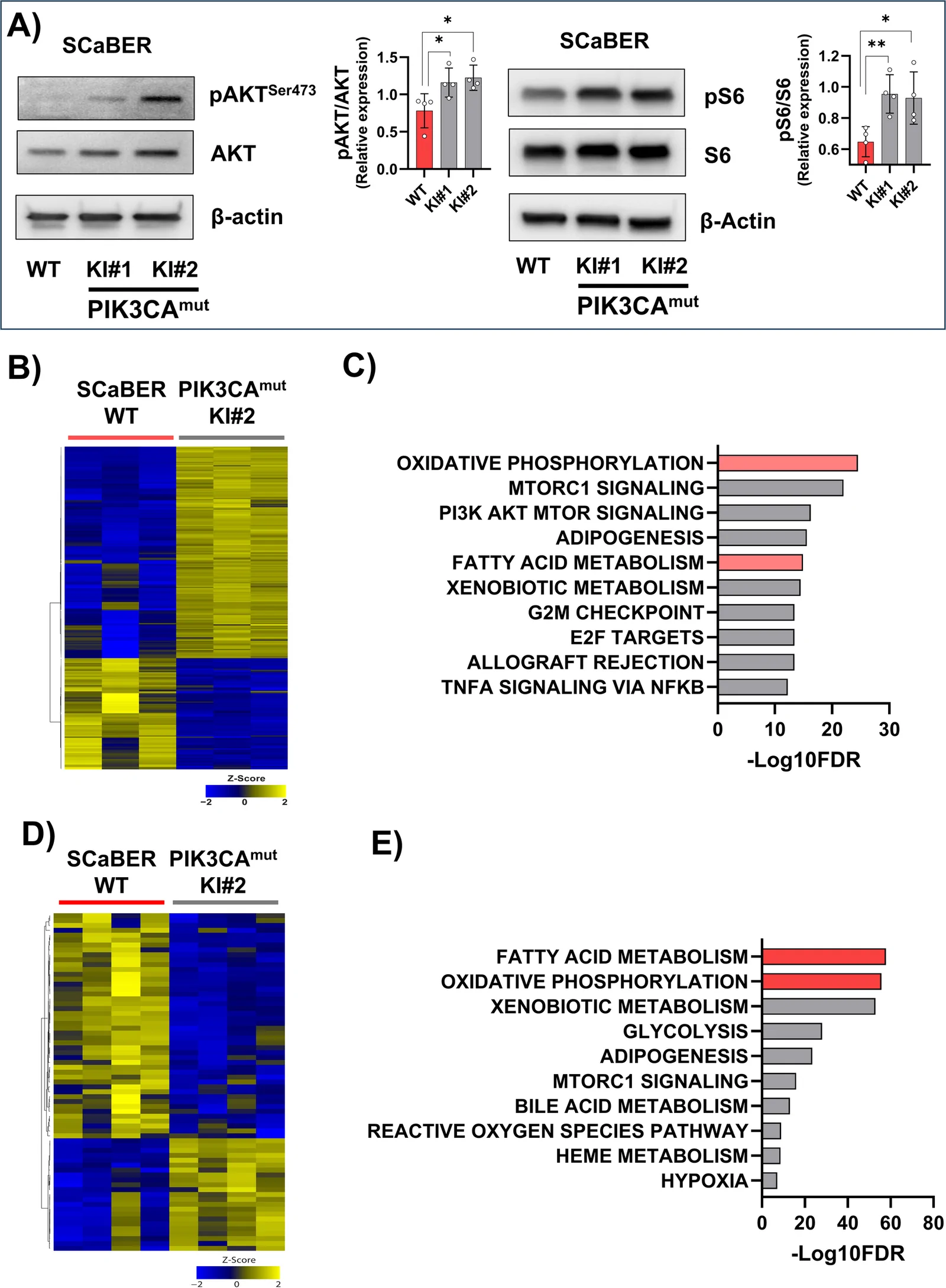
Functional validation and pathway analysis of PIK3CA E545K knock-in models. **A** CRISPR-based knock in of E545K PIK3CA mutation (PIK3CA^mut^) in SCaBER (two independent clones; clone1=KI#1, clone2= KI#2) cell lines with increased pAKTSer473 and pS6 ribosomal proteinSer235/236 confirming functional activity of the knock-in construct. Total AKT and S6 are also measured. Scatter plots represent quantification of pAKT/AKT (n=4) and pS6/S6 (n=4; *P<0.05, **P<0.01). β-actin used for only loading control. **B** Differential heatmap representing metabolic genes comparison between SCaBER WT (n=3) and SCaBER PIK3CA (KI#2) mutant cells (n=3) using nanostring sequencing (FDR<0.25). **C** Top ten significantly enriched Hallmark pathways in SCaBER PIK3CA^mut^ (n=3; KI#2) vs. SCaBER WT (n=3) cells from transcriptomics data (NanoString; refer to supplementary table 03). **D** Heatmap representing altered metabolites comparison between SCaBER WT (n=4) and SCaBER PIK3CA (KI#2) mutant cells (n=4) by targeted metabolomics (FDR<0.25). **E** Top ten Hallmark pathways are significantly enriched in SCaBER PIK3CA^mut^ cells (n=4) compared to SCaBER WT (n=4) cells from metabolomics (refer to supplementary table 06). Differential metabolites (FDR<0.25) were mapped to genes and used for pathway analysis

To establish a human BLCA model for examining the phenotypic outcome of p110α activation, we employed CRISPR/Cas9 genome editing to introduce knock-in (KI) of E545K mutation (PIK3CA^mut^) in SCaBER and UM-UC-3 BLCA cell lines. As shown in earlier studies, *PIK3CA* ^E545K^ mutation promotes cell growth and invasiveness of human cancer cells. The E545K mutation results in an amino acid substitution [26]. With a charge-reversal mutation, the interactions between the protein’s catalytic and regulatory subunits are abrogated, resulting in loss of regulation and constitutive PI3K/AKT signaling pathway [27, 28]. SCaBER and UM-UC-3 BLCA cell lines with an E545K mutation (PIK3CA^mut^) lead to constitutive activation of phospho-AKT levels and pS6 ribosomal protein in two independent clones (KI#1 and KI#2) (Fig. 2A, Supplementary Fig. 1B). Further Sanger sequencing also confirmed the CRISPR/Cas9 based knock-in mutation (E545K) in SCaBER and UM-UC-3 (Supplementary Fig. 1C).

### PIK3CA^mut^ mediated regulation of metabolic pathways in BLCA

In order to determine the effect of PIK3CA^mut^ on oncogenic metabolic rewiring, we performed the targeted transcriptomics analysis (nanoString, nCounter^®^ Metabolic Pathways Panel). This targeted panel has genes involved in core metabolic processes and signaling pathways in the context of cancer. Our analysis revealed 276 differentially expressed genes in SCaBER cell line with PIK3CA^mut^ compared to parental cells (WT cells) (Fig. 2B, Supplementary Table 02). We have identified a set of metabolic genes which are affected by PIK3CA mutations. Our main focus is understanding how PIK3CA^mut^ influences metabolic genes. Enrichment pathway analysis revealed oxidative phosphorylation (OXPHOS) and fatty acid metabolic pathways which are significantly altered by PIK3CA mutation (Fig. 2C, Supplementary Table 03). In addition, gene set enrichment analysis (GSEA) from TCGA BLCA data comparing PIK3CA mutant patients (considering all mutations) versus patients with no PIK3CA mutations revealed positive enrichment of OXPHOS [Normalized Enrichment Score(NES) = 5.63], mTORC1 signaling (NES = 5.10) and fatty acid metabolism (NES = 4.01) in PIK3CA mutant patients (Supplementary Fig. 1D; Supplementary Table 04). PIK3CA activation is associated with coordinated upregulation of OXPHOS, fatty acid metabolism, and mTORC1 signaling gene sets, reflecting enhanced mitochondrial bioenergetics, and anabolic growth programs.

To understand the metabolic phenotype of PIK3CA^mut^ in BLCA, we further evaluated targeted metabolomics using liquid chromatography-mass spectrometry (LC-MS) analysis in BLCA cell lines with and without PIK3CA mutation. Reduced steady-state levels of glutamine, fumarate, and oxaloacetate in PIK3CA-mutant cells suggest enhanced metabolic turnover within mitochondrial and central carbon metabolism. Consistent with this observation, pathway enrichment analysis showed altered OXPHOS, mTORC1 signaling, fatty acid metabolism, xenobiotic metabolism, and adipogenesis pathways in PIK3CA-mutant cells (each cell line with two independent clone) compared with WT cells. (Fig. 2D-E, Supplementary Fig. 2A-F, Supplementary Tables 05–12).

### PIK3CA^mut^ enhances mitochondrial activity via glutamine metabolism

**Fig. 3:**
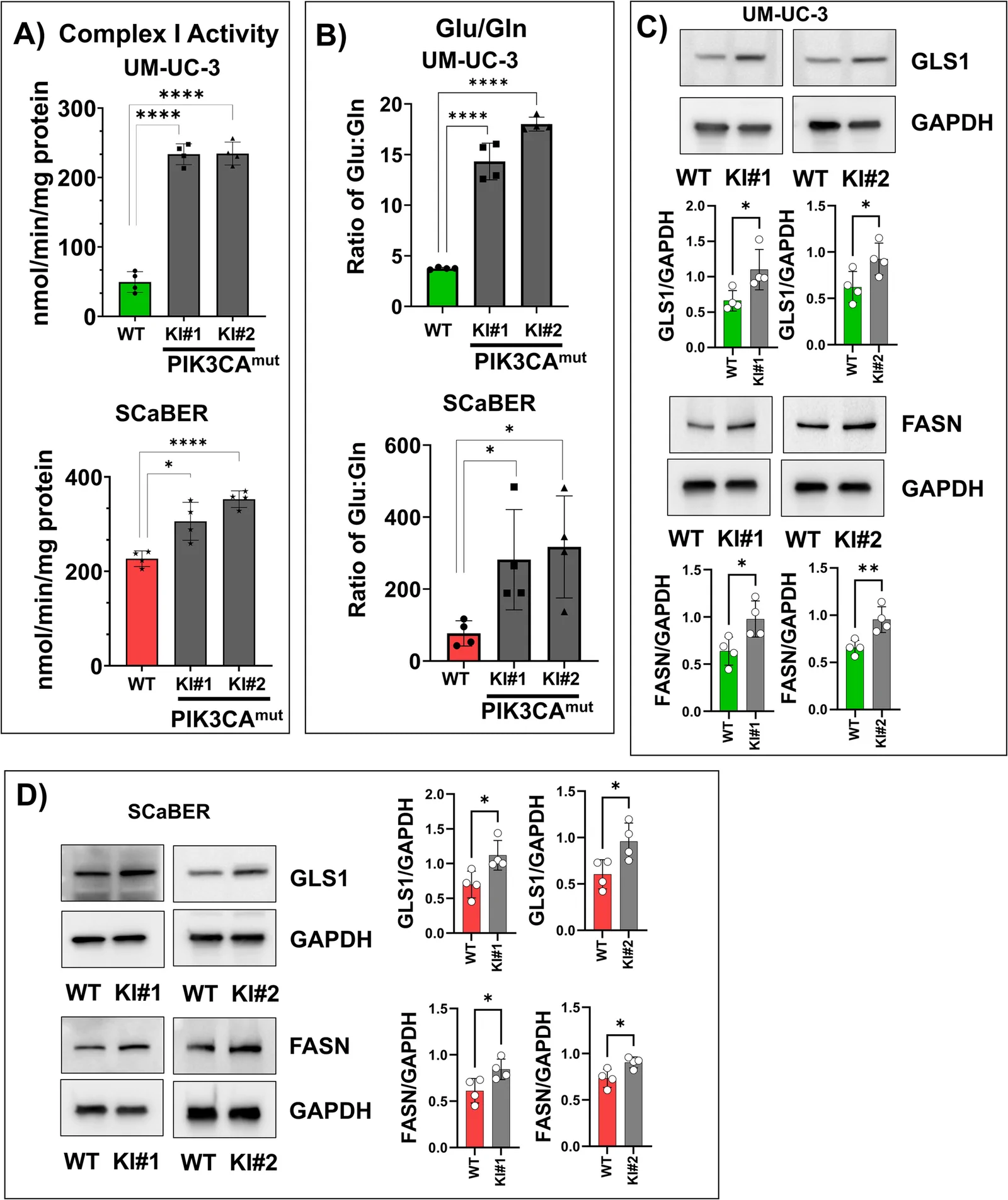
PIK3CA^mut^ drives mitochondrial metabolism via glutamine flux. **A** Mitochondrial complex I activity significantly increased in PIK3CA^mut^ KI#1 and KI#2 compared with WT cells in UM-UC-3 (n=4 per arm) and SCaBER (n=4 per arm) cell line. Optical Density (OD) of complex I activity was measured using specific substrates. (Two biological with two technical replicates were used; *P < 0.05, ****P < 0.0001). **B** Ratio of glutamic acid to glutamine is significantly increased in PIK3CA^mut^ KI#1 and KI#2 vs. WT cells in UM-UC-3 (n=4 per arm) and SCaBER cell lines (n=4 per arm). Peak areas were normalized to internal standard (ISTD) and calculated the ratio of glutamic acid (Glu) to glutamine (Gln). (*P < 0.05, ****P < 0.0001). **C** Immunoblot representing increased expression of GLS1 and FASN in PIK3CA^mut^ KI#1 and KI#2 clones compared to WT in UM-UC-3 cell lines. Quantification of immunoblots for GLS1 (n=4) and FASN (n=4) normalized with associated GAPDH (*P < 0.05, **P < 0.01). **D** Same as C, but for SCaBER cell lines. Quantification of immunoblots for GLS1 (n=4) and FASN (n=4) normalized with associated GAPDH (*P < 0.05)

Transcriptomic and metabolomic pathway analyses identified OXPHOS and fatty acid metabolism as the most consistently altered pathways in PIK3CA^mut^ cells. To validate these findings, we performed electron transport chain (ETC) activity assays, which revealed that PIK3CA^mut^ cells exhibited significantly increased complex I activity compared to WT cells (Fig. 3A). Additionally, activities of other ETC components were also elevated in PIK3CA^mut^ cells relative to WT (Supplementary Fig. 3A-B), indicating enhanced mitochondrial function. Of note, ETC component complex I + III, complex II and citrate synthase activity are consistently elevated in both mutant cell lines compared to WT. Correspondingly, we observed an increase in glutaminolysis, reflected by a higher ratio of glutamic acid to glutamine in PIK3CA^mut^ cells compared to WT cells (Fig. 3B). To further explore this metabolic shift, we measured glutaminase 1 (GLS1), a key enzyme regulating glutamine catabolism, in both UM-UC-3 and SCaBER PIK3CA^mut^ cells. Both cell lines showed markedly elevated GLS1 expression in the two independent knock-in clones, accompanied by increased fatty acid synthase (FASN) levels compared to WT cells (Fig. 3C-D). Together, these results indicate that the PIK3CA E545K mutation drives enhanced glutamine flux into the tricarboxylic acid (TCA) cycle and promotes fatty acid synthesis by supporting mitochondrial complex activity, implicating metabolic reprogramming that may facilitate tumor growth.

### PIK3CA^mut^ drives glutamine flux and alters fatty acid metabolism

**Fig. 4:**
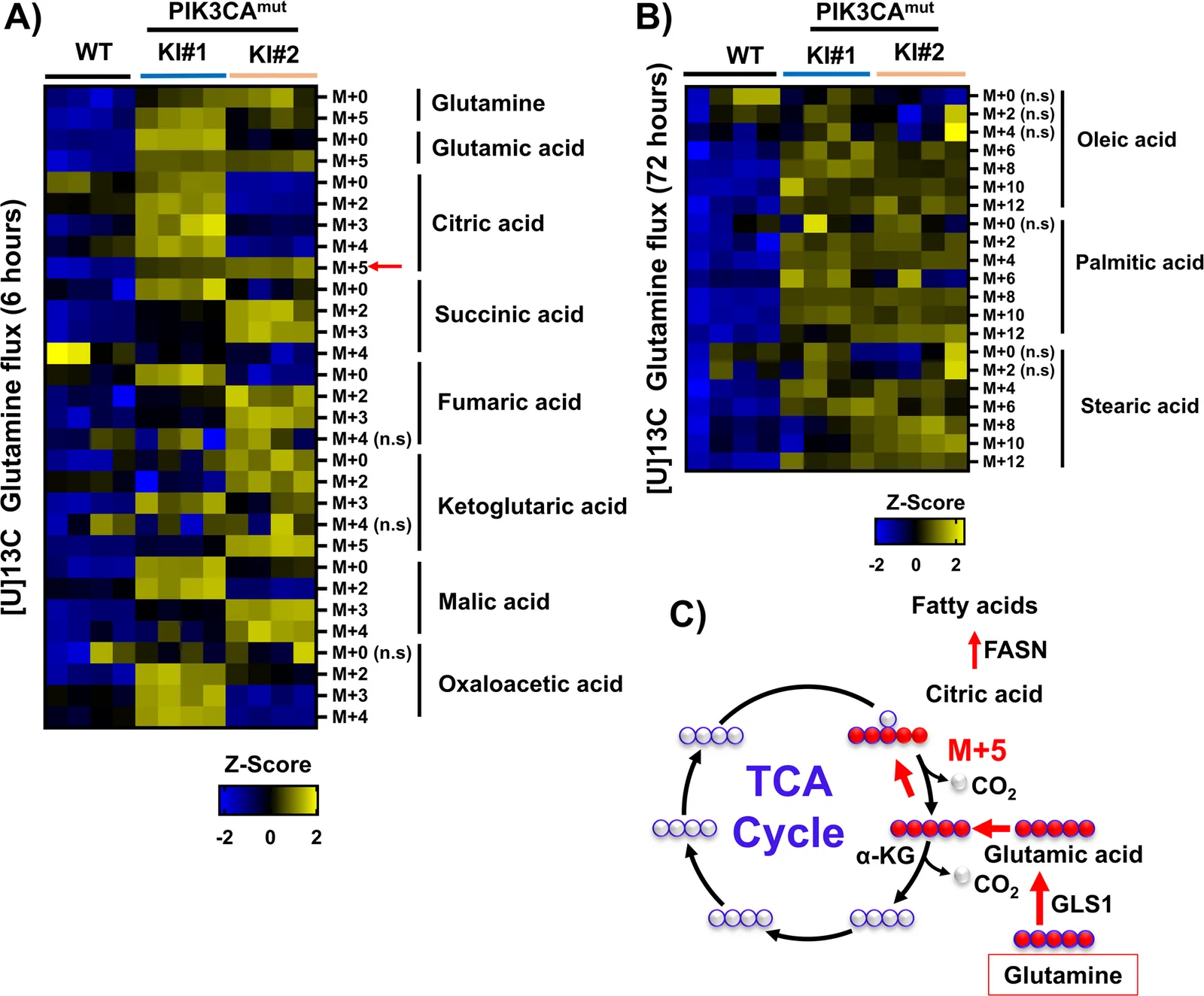
PIK3CA^mut^ enhances glutamine flux into TCA cycle and fatty acids. **A** Heatmap representing [U]13C glutamine incorporation (6 hours) into TCA cycle of SCaBER WT (n = 4), SCaBER PIK3CA^mut^ KI#1 (n = 4) and SCaBER PIK3CA^mut^ KI#2 (n = 4) cell lines. Peak areas are converted to log2 and followed by z score transformation. **B** Heatmap representing [U]13C glutamine incorporation (72 hours) into fatty acids of SCaBER WT (n = 4), SCaBER PIK3CA^mut^ KI#1 (n = 4) and SCaBER PIK3CA^mut^ KI#2 (n = 4) cell lines. Peak areas are converted to log2 and followed by z score transformation. Significance was determined based on log-transformed data and calculated the significance using BH *p* value (FDR) by ANOVA between SCaBER WT, SCaBER PIK3CA^mut^ KI#1 and PIK3CA^mut^ KI#2 (FDR<0.25 otherwise represented n.s =non significance). Yellow represents increased, and blue represents decreased levels from Z score values. **C** Representative diagram showing metabolic flux measurement using glutamine labeled on all its 5 carbons with 13C. Citrate M+5 is a key indicator of reductive carboxylation leading to rapid entry of glutamine-derived carbon into fatty acids

To investigate the impact of PIK3CA E545K mutation on glutamine metabolism, we performed metabolic flux analysis using [U]13C labelled glutamine in SCaBER WT cells and two independent PIK3CA^mut^ knock-in clones. We traced the incorporation of 13 C glutamine flux into TCA cycle intermediates and fatty acids to determine how glutamine contributes to central carbon metabolism. Our analysis revealed that PIK3CA^mut^ cells exhibited increased flux through reductive carboxylation [29–32] via the reverse TCA cycle, resulting in the production of M + 5 citrate, which serve as substrates for enhanced de novo fatty acid synthesis (Fig. 4A-C). Fatty acid synthase (FASN), a key enzyme catalyzing the formation of long-chain fatty acids essential for membrane biogenesis and energy storage, was significantly upregulated in both SCaBER and UM-UC-3 PIK3CA^mut^ clones relative to WT cells (Fig. 3C-D). Targeted metabolomics further confirmed elevated levels of TCA intermediates and fatty acids in PIK3CA^mut^ cells (Supplementary Fig. 4A-B).

To delineate the contribution of canonical versus reductive glutamine metabolism, we performed single-carbon labeling experiments using glutamine labeled at either the first carbon (1-^13^C) or the fifth carbon (5-^13^C). Tracing the 1-^13^C label demonstrated enhanced flux through reductive carboxylation contributing to citrate production, while 5-^13^C labeling indicated active the canonical oxidative TCA pathway (Supplementary Fig. 5A-B). Concurrently, we have observed modest differences in TCA cycle and fatty acid flux in different PIK3CA KI clones. This clonal variation exhibited notable metabolic heterogeneity but consistent with the role of PIK3CA signaling with enhanced mitochondrial metabolism. Collectively, these findings demonstrate that PIK3CA^mut^ cells redirect glutamine-carbon into both the oxidative and reductive arms of the TCA cycle, fueling fatty acid biosynthesis (Supplementary Fig. 5C). This metabolic reprogramming likely supports increased anabolic demands and survival of PIK3CA-mutant BLCA cells.

### PIK3CA^mut^ promotes tumor progression

**Fig. 5:**
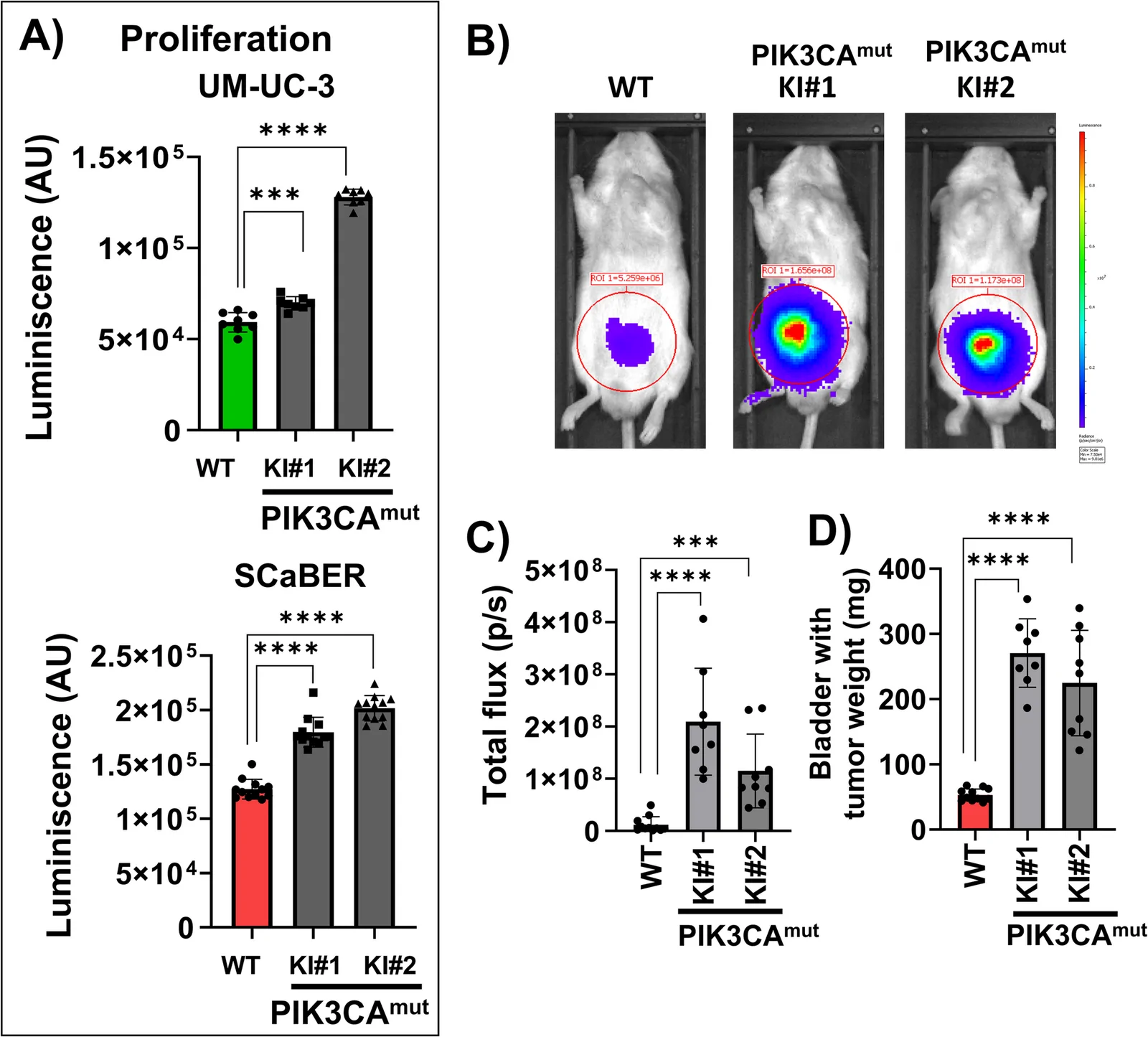
**A** CellTiter-Glo proliferation assay significantly increased in PIK3CA^mut^ KI#1 and KI#2 compared with WT cells in UM-UC-3 (n=8 per arm) and SCaBER (n=12 per arm) cell line. **B** Representative bioluminescence images (BLI) of mice bearing SCaBER (WT), SCaBER PIK3CA^mut^ KI#1, and SCaBER PIK3CA^mut^ KI#2 orthotopic xenografts on week 6. **C** Scatter plot represents the quantification of BLI signal of mice bearing PIK3CA^mut^ KI#1 (n=8), KI#2 (n=9) and WT (n = 11) SCaBER orthotopic xenografts at week 6. **D** Weight of the orthotopic bladder tumors from SCaBER WT (n = 11), PIK3CA^mut^ KI#1 (n=8) and PIK3CA^mut^ KI#2 (n=9) (***P < 0.001, ****P < 0.0001)

To investigate whether the E545K mutant promotes BLCA disease progression, parental cells (WT) or PIK3CA E545K (PIK3CA^mut^) were cultured under identical conditions then we measured the cell growth by cell proliferation assays from UM-UC-3 and SCaBER cell lines. As expected, the cell titer glow assay revealed that cell growth was increased in PIK3CA^mut^ cells (two independent KI clones) as compared with WT cells (Fig. 5A**)**. To validate these observations, we used an orthotopic mouse NOD/SCID/IL2rγnull (NSG) model where luciferase labelled SCaBER human BLCA cells (WT, or PIK3CA^mut^) were injected directly into the bladder wall to establish orthotopic tumors as previously described [33, 34]. We monitored orthotopic tumor growth using bioluminescence imaging (BLI) which showed significantly increased BLI signal in mice bearing PIK3CA^mut^ orthotopic xenograft compared to WT **(**Figs. 5B-C**)**. We observed PIK3CA^mut^ harboring xenografts significantly increased tumor weight compared to WT xenografts (Fig. 5D).

## Discussion

Oncogenic mutations and tumor suppressor loss frequently drive metabolic reprogramming in cancer, coupling genetic alterations to altered nutrient utilization and bioenergetics that support tumor progression [35]. In BLCA, these metabolic adaptations are increasingly recognized as both consequences of driver mutations and potential therapeutic vulnerabilities. Prior studies across multiple tumor types including colorectal, endometrial, breast, and urothelial cancers have consistently shown frequent PIK3CA mutations (ranging from ~ 12% to 46%), underscoring their broad oncogenic role [13]. Our findings provide compelling evidence that BLCA is not a metabolically uniform disease but rather comprises biologically distinct subtypes characterized by various metabolic pathways. These results reinforce the growing consensus that tumor metabolism is context-dependent, influenced by genetic alterations, tumor microenvironment, and host factors. The identification of unique metabolic signatures in specific subtypes provides the potential therapeutic vulnerabilities.

In particular, earlier studies have shown the alteration of energy metabolism in other cancers showing that metabolic plasticity enables tumors to adapt to fluctuations in nutrient availability and immune pressure [36, 37]. Importantly, while prior studies in other cancers [38] [e.g., Head and Neck Squamous Cell Carcinoma (HNSCC), Lung Adenocarcinoma (LUAD), Lung Squamous Cell Carcinoma (LUSC), Liver Hepatocellular Carcinoma (LIHC), and Stomach Adenocarcinoma (STAD)] have shown variability in tumor driver mutations, which are limited for BLCA. Here, we demonstrate that a subset of BLCA patients exhibits a higher frequency of PIK3CA mutations. These results highlight the importance of considering genetic heterogeneity in BLCA biology and may inform the development of mutant specific biomarkers and precision therapies.

Functionally, our preclinical data show that BLCA cells harboring PIK3CA E545K mutations display increased proliferation, tumor growth, and altered mTOR, fatty acid metabolism, and OXPHOS pathways. These findings corroborate prior observations that PIK3CA mutations potentiate oncogenic signaling and metabolic remodeling across multiple cancers [13, 35, 39–42]. Metabolically, our findings reveal that PIK3CA-mutant BLCA cells undergo profound metabolic rewiring characterized by increased glutamine flux. Mutant cells preferentially channel glutamine through reductive carboxylation to sustain TCA cycle activity, which is coupled with enhanced mitochondrial complex I function and increased fatty acid biosynthesis, consistent with elevated GLS1 and FASN expression. PIK3CA mutations enhances anabolic and energetic demand which may activates various transcription factors such as ATF4 which will regulate the GLS1 expression under metabolic stress [43]. Further mechanistic studies are required to validate GLS1-ATF4 axis in the context of PIK3CA mutant BLCA progression.

Supporting this, steady-state and 13 C glutamine flux metabolomic profiling demonstrated increased levels of TCA cycle intermediates and fatty acid metabolites, indicating enhanced mitochondrial metabolic activity in PIK3CA mutant cells. Furthermore, 13 C-glutamine tracing revealed increased one-carbon glutamine flux into citrate and downstream metabolites through the reductive carboxylation pathway, providing direct evidence that PIK3CA mutations drive increased glutamine flux and energy metabolism. Collectively, these data establish glutamine-driven reductive metabolism as a key metabolic adaptation that supports bioenergetic and biosynthetic demands in PIK3CA-mutant BLCA cells.

In summary, our findings suggest that PIK3CA mutations contribute to the elevated oxidative phosphorylation and glutamine metabolism observed in BLCA, potentially explaining disease progression and prognosis. While the proliferative advantage and PI3K inhibitor sensitivity observed in PIK3CA-activated models are consistent with established biology, accumulating evidence indicates that metabolic adaptations may influence the cellular response to PI3K pathway inhibition [19, 44]. In line with this, our transcriptomic and metabolomic analyses suggest altered mitochondrial metabolic activity in PIK3CA-driven tumors, highlighting a potential avenue for rational combination therapy. To enhance the impact of these results, future studies could be implemented by evaluating combinatorial strategies that pair PIK3CA-targeted therapies with metabolic inhibitors.

These insights support the development of strategies combining PIK3CA-targeted therapies with metabolic inhibitors, particularly those affecting mitochondrial function or glutaminolysis. Further mutational and metabolic profiling of BLCA tumors will be essential to fully understand the interplay between genetic drivers and metabolic rewiring and to enable biomarker-driven precision therapies for BLCA.

While our study highlights potential metabolic and signaling alterations, several mechanistic conclusions remain preliminary due to sample power and prevalence of mutations which is a limitation in the current study. Future studies should validate PIK3CA and other mutations in larger BLCA cohorts and employ comprehensive genomic and metabolomic profiling to uncover additional alterations and metabolic rewiring. Integration of single-cell analyses could clarify tumor heterogeneity, while testing combinatorial targeted therapies in patient-derived models will be essential to assess long-term efficacy and support precision medicine approaches. Mitochondrial dysfunction by PIK3CA mutation was highlighted in current study and there could be other potential alterations that may be driven by mutations which will be studied in future.

## Materials and methods

### Sex as a biological variable

This study included both male and female human bladder cancer (BLCA) samples. For mouse experiments, only male animals were used to assess tumor growth; therefore, sex was not incorporated as a biological variable.

### Clinical samples

For this study, human BLCA tissues were collected from Augusta University (AU), and University of Texas Southwestern Medical Center (UTSW) obtained under protocols approved by the respective Institutional Review Board (IRB) and stored at − 140 °C. Samples used for this study and clinical information was provided in Supplemental Table 1. All the samples were obtained with written informed consent and were de identified before analysis. 

### Targeted amplicon sequencing (AmpliSeq for Illumina Cancer Hotspot Panel v2)

Genomic DNA was extracted from bladder cancer tissues (fresh-frozen or FFPE) using the Qiagen DNA isolation kit under standard conditions. DNA concentration was determined with a Qubit dsDNA HS assay, and quality was assessed using TapeStation or Bioanalyzer. When possible, 10 ng of DNA per sample was used for library construction, with a minimum input of 1 ng for high-quality DNA. Targeted sequencing libraries were prepared with the AmpliSeq for Illumina Cancer Hotspot Panel v2, covering 50 genes with recurrent hotspot mutations. Multiplex PCR amplification of hotspot regions was followed by partial primer digestion and ligation of index adapters. Libraries were cleaned with AMPure XP beads, quantified with Qubit, and evaluated for expected fragment size (~ 200–275 bp with adapters). Normalized libraries were pooled and sequenced on the Illumina NextSeq 2000 platform with 2 × 150 bp paired-end reads using the standard workflow. Sequencing performance metrics (e.g., cluster density, %≥Q30) satisfied Illumina quality thresholds. Demultiplexing was carried out with bcl2fastq, DRAGEN, or BaseSpace software. Reads were aligned to the human reference genome (hg19 or GRCh38) with BWA-MEM or DRAGEN. Variant detection was performed using tools optimized for amplicon sequencing (e.g., DRAGEN Tumor-Only, Mutect2, or VarDict) with parameters to soft-clip primer sequences and apply base and mapping quality filters. Detected variants were annotated with VEP or ANNOVAR and cross-referenced with COSMIC, ClinVar, and gnomAD databases. Variants with gnomAD allele frequency > 0.1% in non-cancer populations were excluded unless biologically relevant. The assay’s depth allowed for detection of low-frequency variants; a conservative threshold of 2–5% variant allele frequency was applied with additional filters for sequencing depth and strand bias. Samples for mutational analysis were performed in two batches as described in Fig. 1.

### Generation of PIK3CA E545K knock-in cell lines

*CRISPR-Cas9 Guide RNA Design.* To generate the E545K point mutation in the PIK3CA locus, two candidate guide RNAs were designed to target sequences surrounding the E545 codon.

- gRNA1: UcUcUcUgaaaUcacUgagc
- gRNA2: UgcUcagUgaUUUcagagag

Cleavage efficiency analysis demonstrated that gRNA1 was more efficient, and it was selected for knock-in experiments. *Knock-In Donor Template Design.* A single-stranded oligodeoxynucleotide (ssODN) donor template (reverse complement) was designed based on the gRNA1 target site. The ssODN sequence was: 5′-tctccattttagcacttacctgtgactccatagaaaatcCttctcTtgTtTagtgatttcagagagaggatctcgtgtagaaattgctttgagctgttct-3′. This donor template incorporates the E545K point mutation (T & T), a silent mutation introducing an HpyAv restriction site (C), and a silent mutation disrupting the PAM sequence to prevent Cas9 re-cleavage (T). *Primer Design for Genomic PCR and Sequencing.* Primers flanking the E545 region were designed for genomic PCR amplification and knock-in verification: PCR forward: 5′-tcatctgtgaatccagaggggaaa, PCR reverse: 5′-ggcaaagaacacaaaaggaaaatgc. For sequencing of knock-in alleles, the following primer was used: Seq reverse: 5′-tgtgccaactaccaatgtag. *Electroporation and Cell Recovery.* BLCA cells were electroporated with Cas9/sgRNA ribonucleoprotein (RNP) complexes and the ssODN donor using the Neon electroporation platform (Thermo Fisher Scientific). After electroporation, cells were cultured in complete medium for 48 h and then plated at single-cell density (1 cell/well) in 96-well plates. *Clonal Expansion and Screening.* Individual colonies were expanded into 48-well plates. Genomic DNA was extracted, PCR amplified and screened by HpyAv digestion. Colonies displaying complete digestion, indicative of homozygous knock-in, were further confirmed by Sanger sequencing of the undigested PCR products.

### Nanostring nCounter^®^ metabolic pathways panel analysis

Total RNA was extracted from bladder cancer cell lines using the RNeasy Mini Kit (Qiagen) according to standard protocols. RNA quality and quantity were evaluated with an Agilent 2100 Bioanalyzer and a Qubit Fluorometer (Thermo Fisher Scientific). For NanoString nCounter analysis, 100 ng of RNA with integrity scores above 7.0 was hybridized with probes from the nCounter^®^ Metabolic Pathways Panel (NanoString Technologies) at 65 °C for 16–18 h. The hybridized products were subsequently processed on the nCounter Prep Station, which purifies and immobilizes probe–target complexes onto cartridges. Data collection was carried out on the nCounter Digital Analyzer at maximum resolution (555 fields of view per sample). Raw counts underwent quality assessment and normalization using nSolver™ Analysis Software (NanoString Technologies), with background correction based on negative controls and expression levels adjusted relative to internal housekeeping genes.

### Metabolomics analysis

#### Sample preparation

Targeted metabolomics was performed as described previously [34, 45, 46]. Briefly, Metabolites were extracted from BLCA cell lines (3 million cells), and mouse liver. Pooled samples of mouse liver were used for quality control. *LC-MS data review and analysis.* The data was analyzed using Agilent Mass Hunter Quantitative Analysis software (Agilent Technologies).

#### Statistical analysis for metabolomics

The data was log_2_ transformed along with internal standard normalization on a per-sample, per-method basis. For every metabolite in the normalized data set, 2-tailed *t* tests were conducted to compare expression levels between the groups. Differential metabolites were identified by adjusting the *P* values for multiple testing at an FDR threshold of < 0.25. The R statistical software system generated a hierarchical cluster of the differentially expressed metabolites (https://www.r-project.org/).

#### Pathway analysis

Differentially expressed metabolites were mapped to genes using HMDB database. Each metabolite was linked to genes encoding enzymes known to catalyze its synthesis, degradation, or direct interconversion, based on enzyme commission (EC) numbers and reaction-level annotations. Only experimentally validated or high-confidence curated associations were retained. Metabolites without annotated enzymatic reactions were excluded from gene-level analyses.

For metabolites associated with multiple enzymatic reactions or enzymes encoded by multiple genes, all corresponding genes were retained and treated as independent contributors to the metabolite-associated gene set. Gene sets were subsequently collapsed at the pathway level using curated pathway definitions from multiple databases.

To ensure robustness, pathway-level enrichment analyses were performed using gene-level aggregation rather than individual metabolite counts. This approach minimizes bias introduced by highly connected metabolites or multifunctional enzymes and enables consistent integration with transcriptomic and proteomic datasets. Using mapped genes, we performed pathway analysis with python 2.7. As described above, Hypergeometric Enrichment analysis was conducted to identify key hallmark pathways.

### Cell culture and treatments

UM-UC-3 and SCaBER (HTB-3) cell lines were purchased from ATCC, All BLCA cells were grown in stable MEM as described previously [34].

#### 13 C metabolic flux using all carbon labeled [U]13 C-glutamine and single carbon labeled glutamine

Equal number of cells were plated in 10 cm dishes using stable MEM (0.4344 g/L stable glutamine, 1 g/L glucose, and no sodium pyruvate) supplemented with 10% FBS and 1× penicillin-streptomycin cultured overnight at 37 °C under 5% CO_2_. Following day, cells were switched to glutamine-free (no glutamine, 1 g/L glucose) media containing 10% dialyzed FBS for 2 h, and subsequently incubated with 4mM [U]13 C-glutamine in glutamine-free media along with dialyzed FBS. Six hours of incubation were used for TCA metabolite measurement and 72 h of incubation is used for fatty acids measurement. After incubation, the medium was removed, and the cells were washed 3 times with cold PBS. Extraction procedure for the metabolomic analysis is similar to targeted metabolomics. Similar to targeted metabolomics, TCA and fatty acids flux were measured as described in earlier publications [34].

#### Metabolic flux analysis

For metabolic flux and the incorporation measurements, the peak areas for the labeled and unlabeled metabolites were transformed into log2. Anova BH *p* value (FDR) are calculated. Following the log transformation, we used *Z* score transformation of the data represented as a heatmap for all the labeled and unlabeled metabolites.

### Western blot analysis

Western blotting was performed as described [34]. BLCA cells were lysed in RIPA buffer containing protease and phosphatase inhibitors, and protein concentrations were quantified using the BCA assay. Equal protein amounts were separated by SDS-PAGE, transferred to PVDF membranes, and probed with primary and HRP-conjugated secondary antibodies. Signals were detected with the Bio-Rad ChemiDoc XRS system. Antibodies are listed in Supplemental Table 13. All blots were performed in at least four independent biological replicates. Quantification was performed using ImageJ 1.54 g software.

### CellTiter-Glo proliferation assay

Cell proliferation was measured using the Promega CellTiter-Glo assay according to the manufacturer’s instructions. BLCA cells (5,000/well) were plated in 96-well white plates and cultured in MEM supplemented with 10% FBS. After removing ~ 90 µL of medium, cells were incubated with CellTiter-Glo reagent for 10 min, and luminescence was recorded using a Tecan Infinite M200 plate reader. Luminescence values, proportional to cell numbers, were plotted as arbitrary units (AU) in GraphPad Prism v11.0.2, and statistical significance was determined by t test.

### ETC analysis

BLCA cells (5 × 10⁶) were lysed in cell lysis buffer and sonicated at 4 °C using a Qsonica sonicator. Protein concentrations were determined by the Bradford assay for normalization. ETC enzyme activities were measured in two biological with two technical replicates on a Tecan Infinite M200, as previously described [34]. Data was plotted in GraphPad Prism v11.0.2, and statistical significance was assessed by t-test.

### In vivo studies

NSG mice (4–6 weeks of age, male mice) were purchased from The Jackson laboratory. Orthotopic mice experimental details were described in an earlier publication [33, 34]. The body weight of the mice in all the experiments was measured weekly once.

### Reagents used for this study

All the antibodies, compounds, and other chemicals/reagents used for this study are listed in Supplemental Table 13.

### Statistical analysis

Statistical significance was determined for two-sided analysis at a significance level of *P* < 0.05. GraphPad Prism v11.0.2 was utilized for graph analysis, while R software and GraphPad Prism v11.0.2 were employed for heat map generation. Error bars depict standard deviation (SD), or standard error of the mean (SEM) as specified.

## Supplementary Information

Supplementary Material 1. Supporting Data 1.

Supplementary Material 2. Supplementary Tables

Supplementary Material 3. Supplementary Figures

Supplementary Material 4. Supporting Data 2.

## Data Availability

All the raw data was available in supporting data. Raw data for the mutation sequencing are uploaded to sequence read archive (SRA) with submission ID for batch1 PRJNA1432942 and batch2 PRJNA1433030. Raw and processed nanostring RNA Sequence data from cell lines are uploaded at GEO Hub (GSE323369). Raw and processed metabolomics data from samples are available at Metabolomics workbench ID: ST004218.
