# Supplementary material for: Oncogenic PIK3CA reprograms glutamine metabolism to drive bladder cancer progression
Source: Cell Commun Signal. 2026 Jul 14;24:562. doi: 10.1186/s12964-026-03058-w (PMC13679264; doi:10.1186/s12964-026-03058-w)
Supplement: Supplementary file 1 [file 12964_2026_3058_MOESM3_ESM.pdf]

Supplementary Figures (1-5)

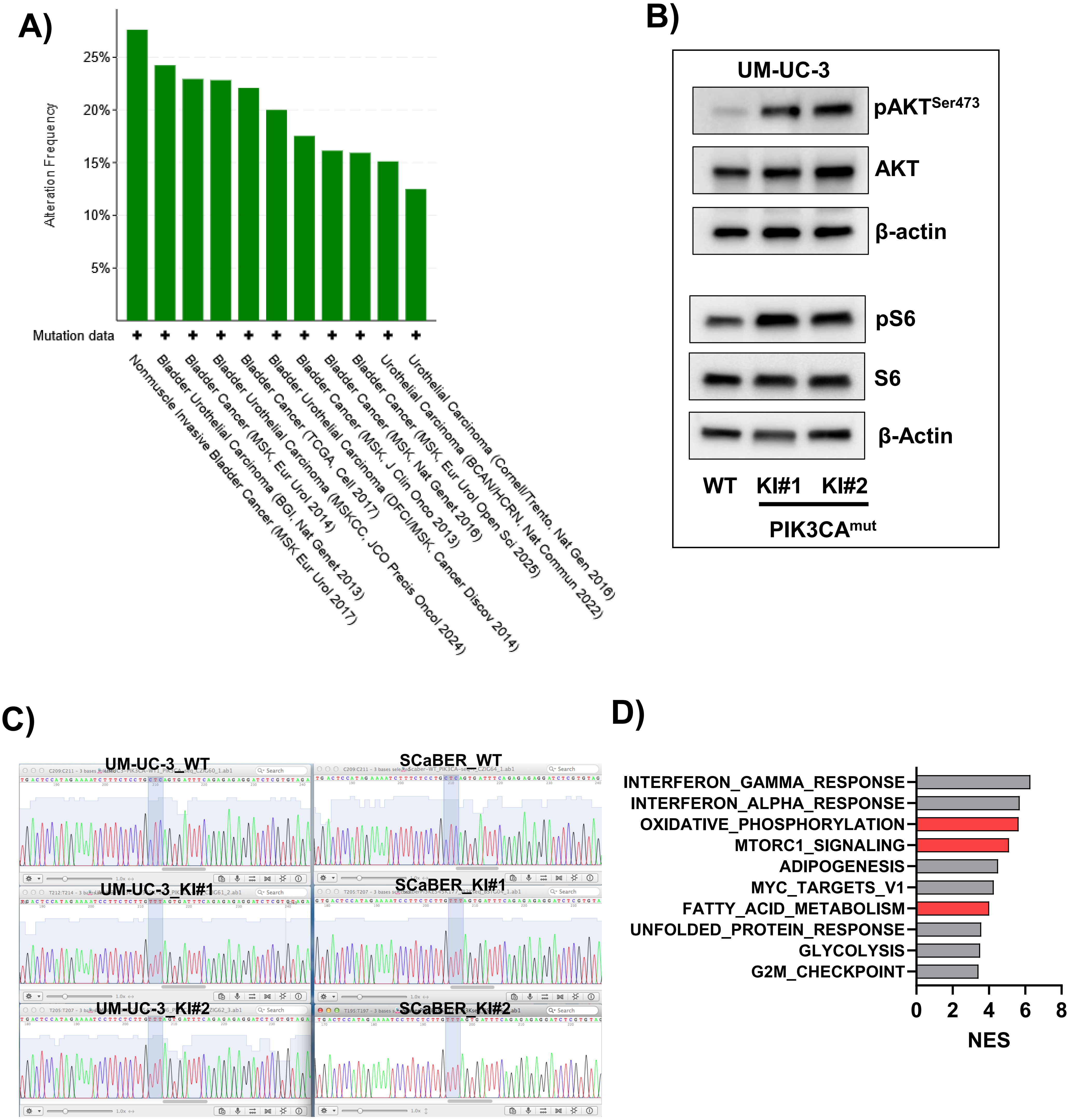

Supplementary Figure 01. A) Frequency of PIK3CA alterations in multiple bladder cancer cohorts. (Image obtained from cBioportal). **B)** CRISPR-based knock in of E545K PIK3CA mutation (PIK3CA^{mut}) in UM-UC-3 (two independent clones; clone1=KI#1, clone2= KI#2) cell lines with increased p-AKT^{Ser473} and pS6 ribosomal protein$^{Ser235/236}$ confirming functional activity of the knock-in construct. Total AKT and S6 ribosomal protein are also measured. β-actin used as a loading control. **C)** CRISPR-based knock in confirmation of GAG (Glutamic acid) to AAA (Lysine) in UM-UC-3 and SCaBER BLCA cell lines by sanger sequencing. **D)** Bar graph representing top 10 positively enriched pathways (refer to supplementary table 04) from patients with PIK3CA mutations (n=90) compared to patients with no PIK3CA mutations (n=318) from TCGA BLCA.

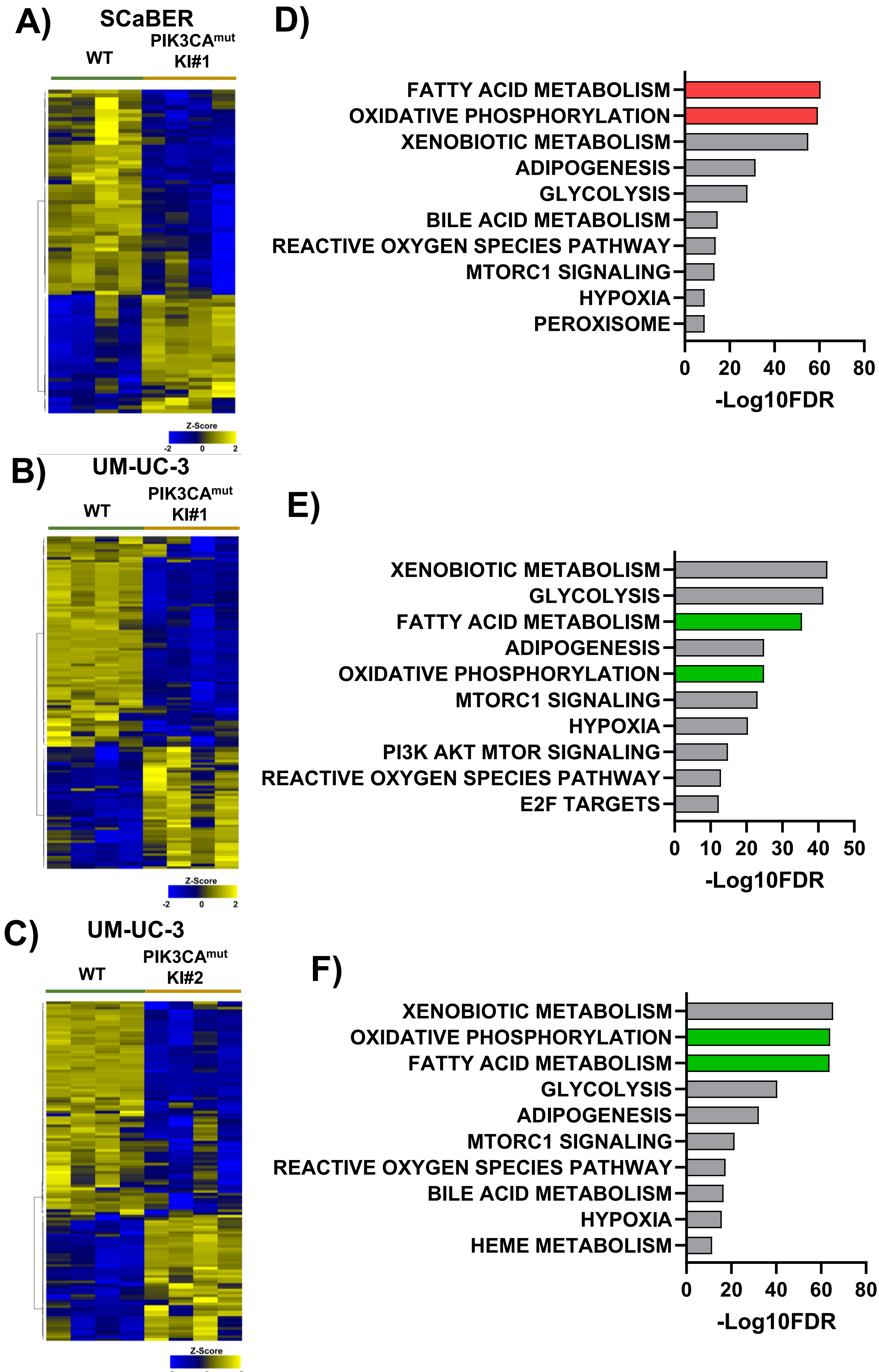

Supplementary Figure 02. Metabolic alteration in PIK3CA mutant BLCA cell lines. A) Differential Heatmap representing altered metabolites comparison between SCaBER WT (n=4) and SCaBER PIK3CA (KI#1) mutant cell (n=4) by targeted metabolomics (FDR<0.25)**. B)** Differential Heatmap representing altered metabolites comparison between UM-UC-3 WT (n=4) and UM-UC-3 PIK3CA (KI#1) mutant cell (n=4) by targeted metabolomics (FDR<0.25). **C)** Differential Heatmap representing altered metabolites comparison between UM-UC-3 WT (n=4) and UM-UC-3 PIK3CA (KI#2) mutant cell (n=4) by targeted metabolomics (FDR<0.25). **D-F)** Representing top ten Hallmark pathways are significantly enriched in PIK3CA mutant cells (SCaBER KI#1, UM-UC-3 KI#1, UM-UC-3 KI#2) compared to WT cells (SCaBER, UM-UC-3) from metabolomics. Differential metabolites were mapped to genes and used for pathway analysis (Refer to supplementary tables 8, 10 and 12).

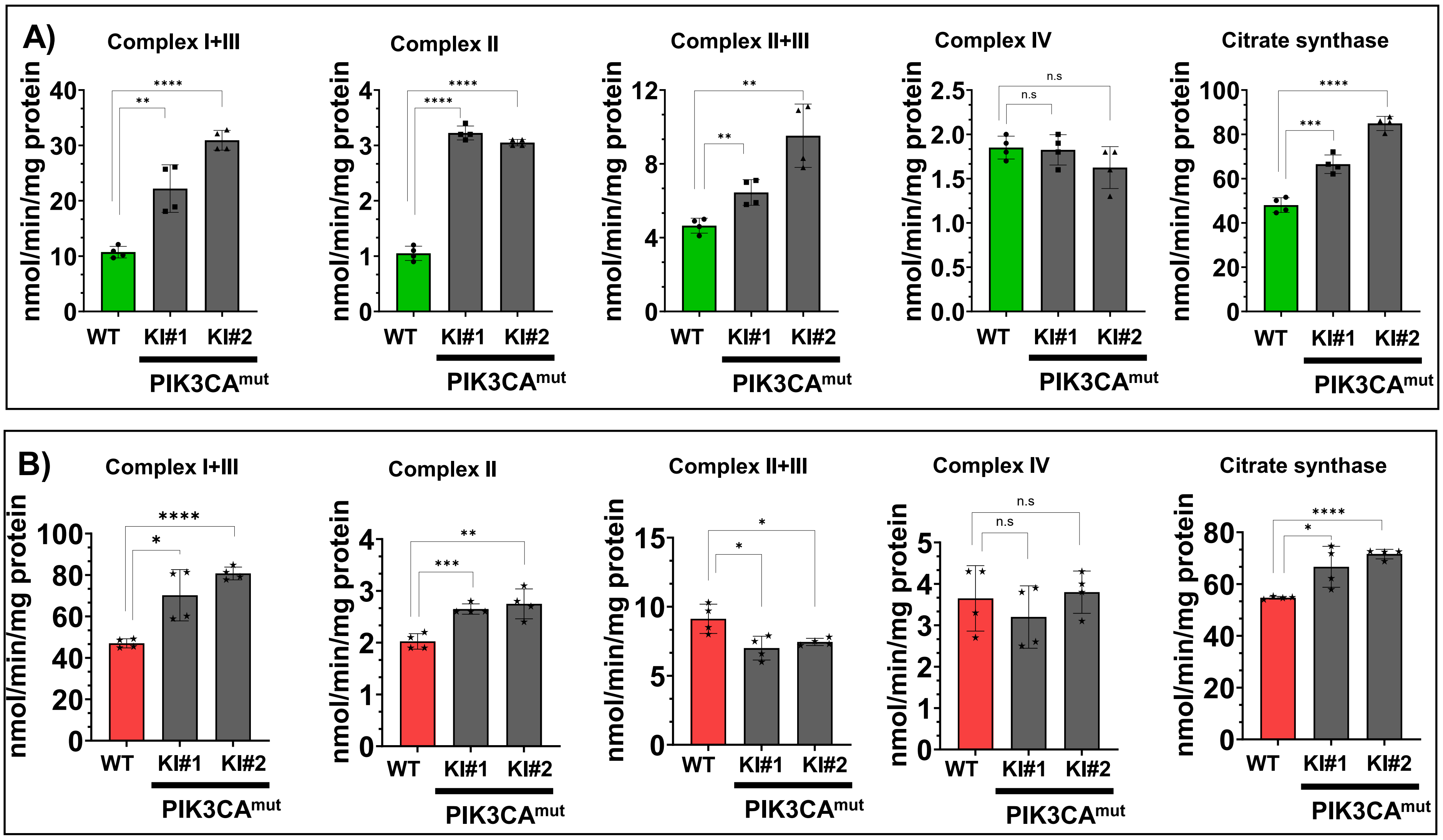

Supplementary Figure 03. A) Scatter plots representing the differential mitochondrial complex activities in PIK3CA^{mut} cells (UM-UC-3 KI#1, KI#2) compared to WT(UM-UC-3) cells. **B**). Scatter plots representing the differential mitochondrial complex activities in PIK3CA^{mut} cells (SCaBER KI#1, KI#2) compared to WT(SCaBER) cells. OD of complex activity was measured using specific substrates which was measured by colorimetric assay and normalized with protein concentration. (Two biological analyzed under two technical replicates, n=4; *P < 0.05, **P < 0.01, ***P < 0.001, ****P < 0.0001, n.s represents non significance).

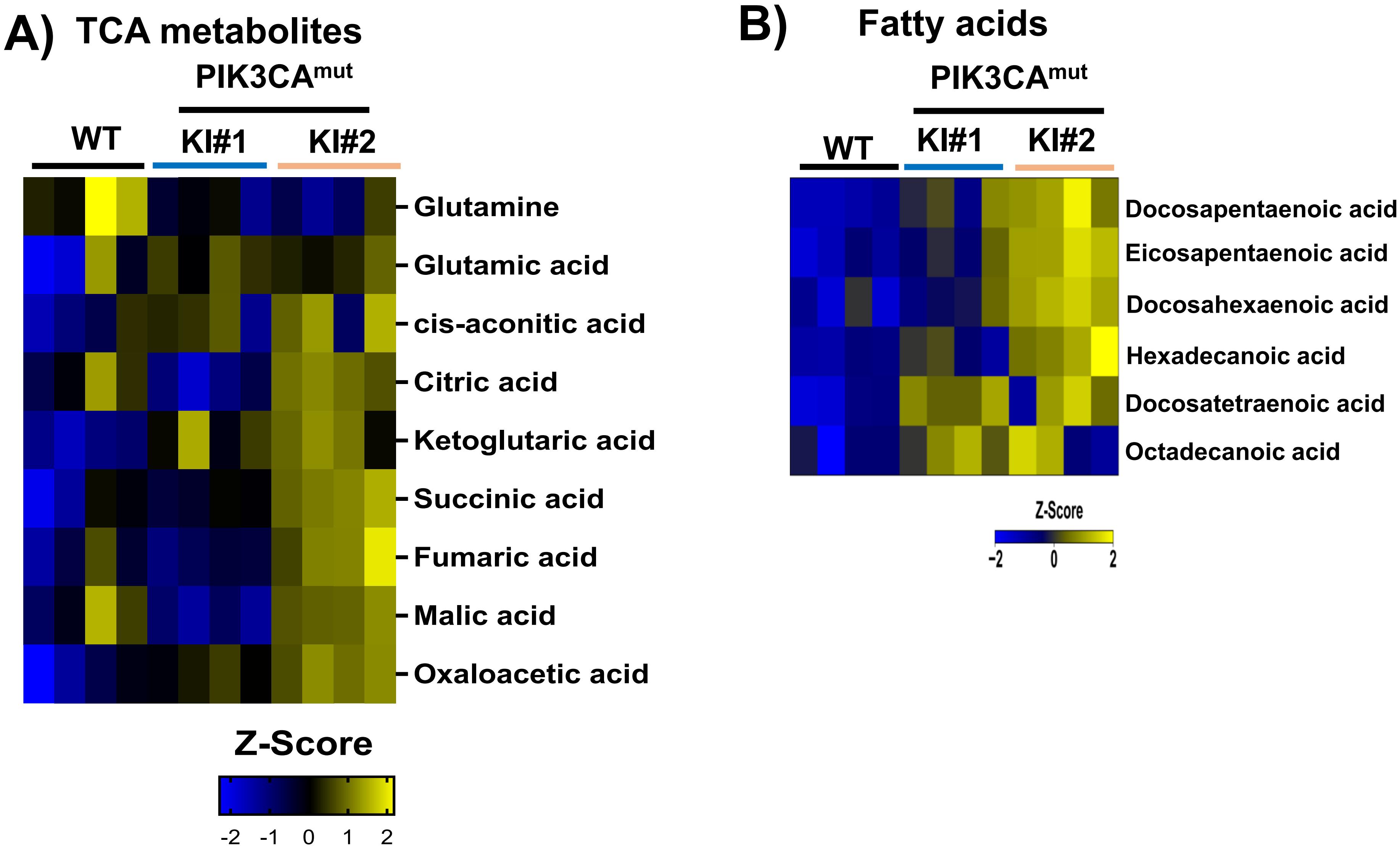

Supplementary Figure 04. A) Heatmap represents the steady state levels of TCA metabolites (FDR <0.25) in PIK3CA^{mut} cells (two independent clones) and SCaBER WT cells (n=4 per arm). **B)** Heatmap represents the steady state levels of fatty acids (FDR <0.25) in PIK3CA^{mut} cells (two independent clones) and SCaBER WT cells (n=4 per arm).

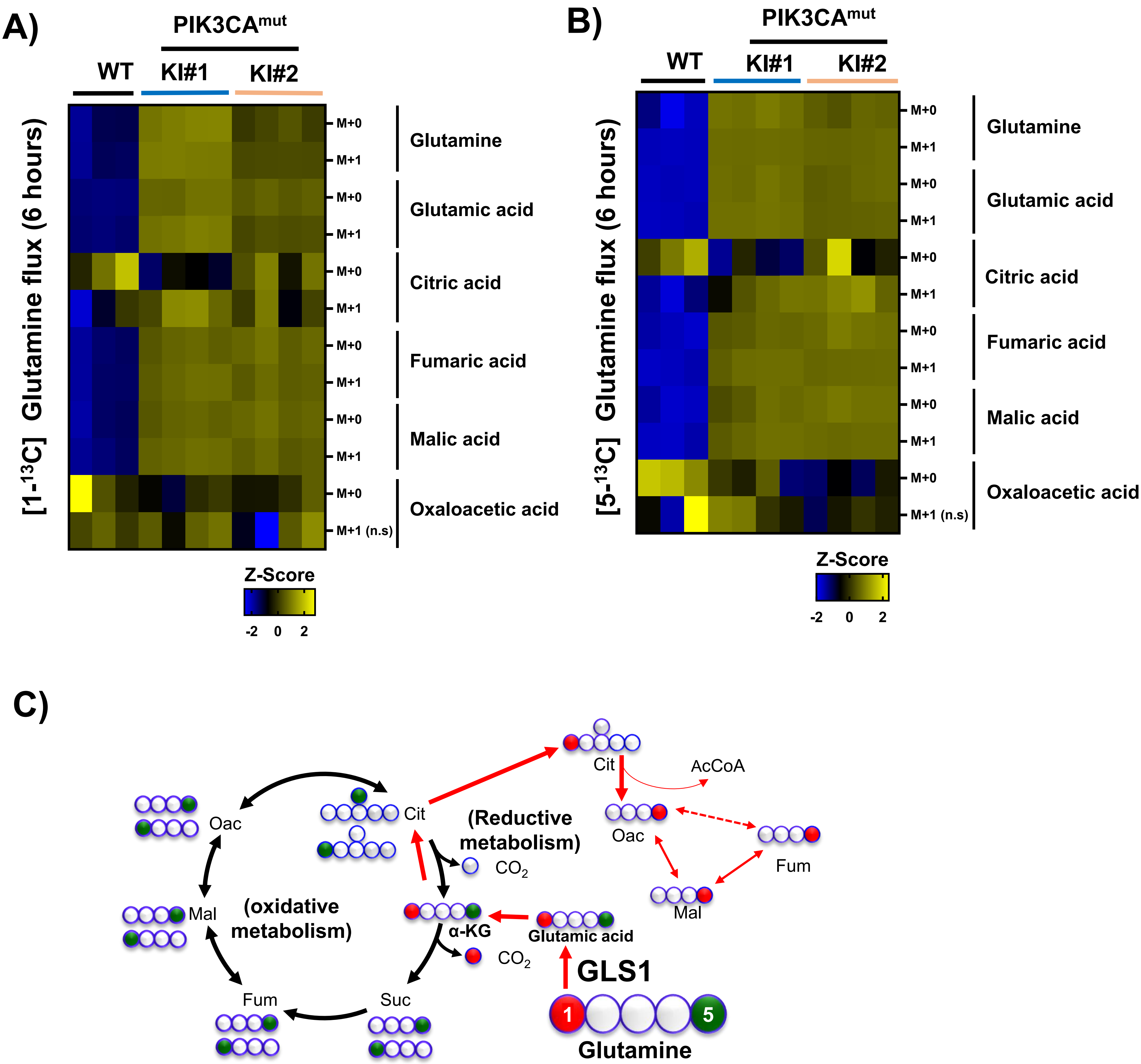

Supplementary Figure 05. A) Heatmap represents the [1-^{13}C] glutamine labeled carbon into TCA metabolites (6 hours) in PIK3CA mutant cells (SCaBER KI#1, KI#2; n=4 replicates for each clone) and SCaBER WT cells (n=3). **B)** Heatmap represents the [5-^{13}C] glutamine labeled carbon into TCA metabolites (6 hours) in PIK3CA mutant cells (SCaBER KI#1, KI#2; n=4 replicates for each clone) and SCaBER WT cells (n=3). Peak areas are converted to log2 and followed by z score transformation. Significance was determined based on log-transformed data and calculated the significance using BH p value by anova between SCaBER WT, SCaBER PIK3CA^{mut} KI#1 and PIK3CA^{mut} PIK3CA KI#2 (Panel A and B metabolites with FDR<0.25 otherwise represented as n.s =non significance). Yellow represents increased, and blue represent decreased levels from Z score values. **C)** Representative schematic diagram showing metabolic flux measurement using 13C labeled glutamine on its 1st (α-carboxyl carbon) and 5th carbon (amide carbon) into TCA cycle highlighting the reductive carboxylation and canonical oxidation, respectively (Cit = Citric acid; α-KG = α-Ketoglutaric acid; Suc = Succinic acid; Fum = Fumaric acid; Mal = Malic acid; Oac = Oxaloacetic acid).
