# Supplementary material for: Oncogenic PIK3CA reprograms glutamine metabolism to drive bladder cancer progression
Source: Cell Commun Signal. 2026 Jul 14;24:562. doi: 10.1186/s12964-026-03058-w (PMC13679264; doi:10.1186/s12964-026-03058-w)
Supplement: Supplementary file 2 [file 12964_2026_3058_MOESM4_ESM.pdf]

Title: Oncogenic PIK3CA reprograms glutamine metabolism to drive bladder cancer progression.

Supporting data 2 associated with manuscript

Unedited blots associated with Figure 2A_SCaBER_left panel

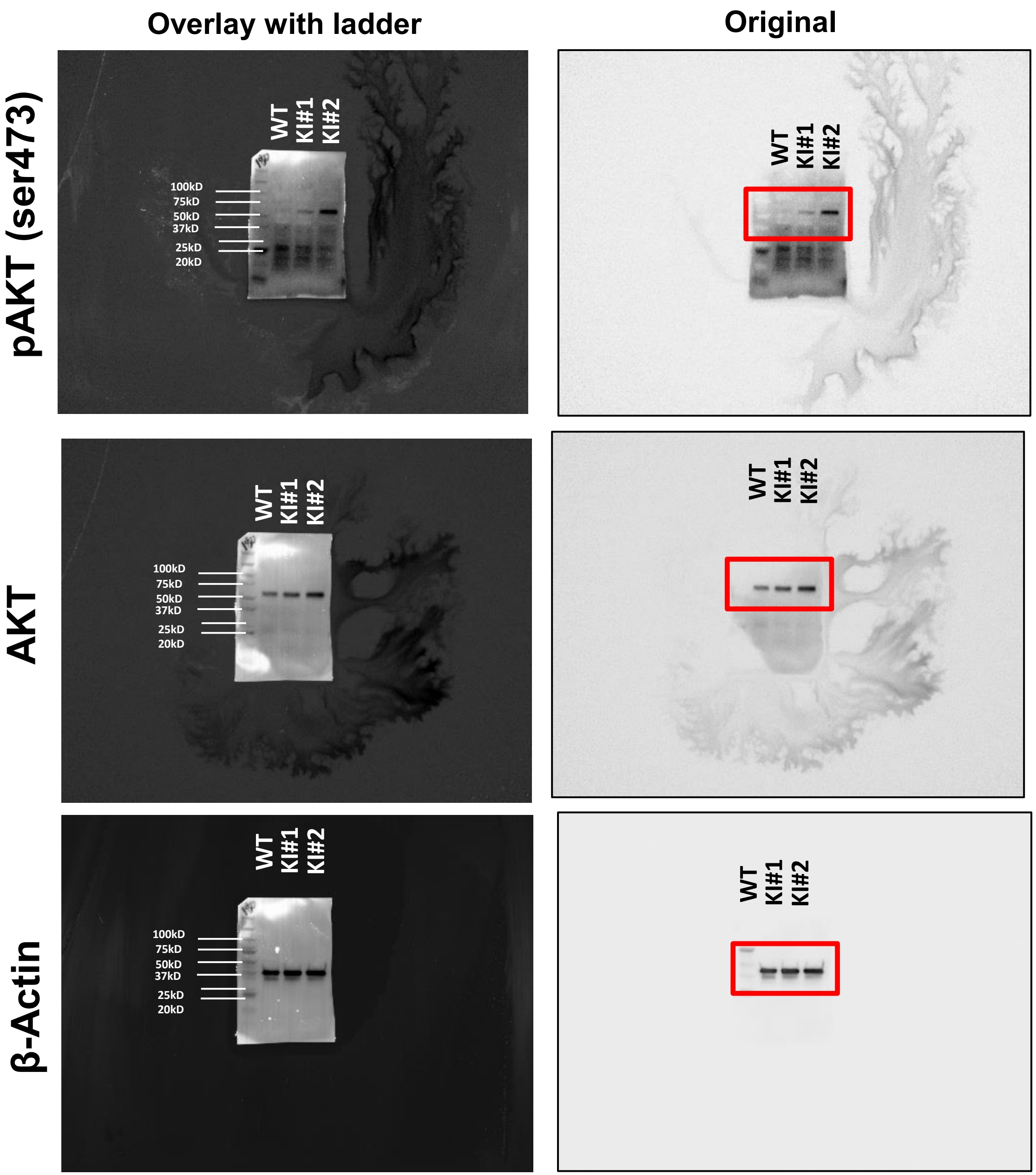

Area used for assembled the figure

pAKT ser473
Predicted mol. Wt: 60kDA
Observed mol. Wt: 60kDA
AKT
Predicted mol. Wt: 60kDA
Observed mol. Wt: 60kDA

Blot no:180

Unedited blots associated with Figure 2A_SCaBER_ Right panel

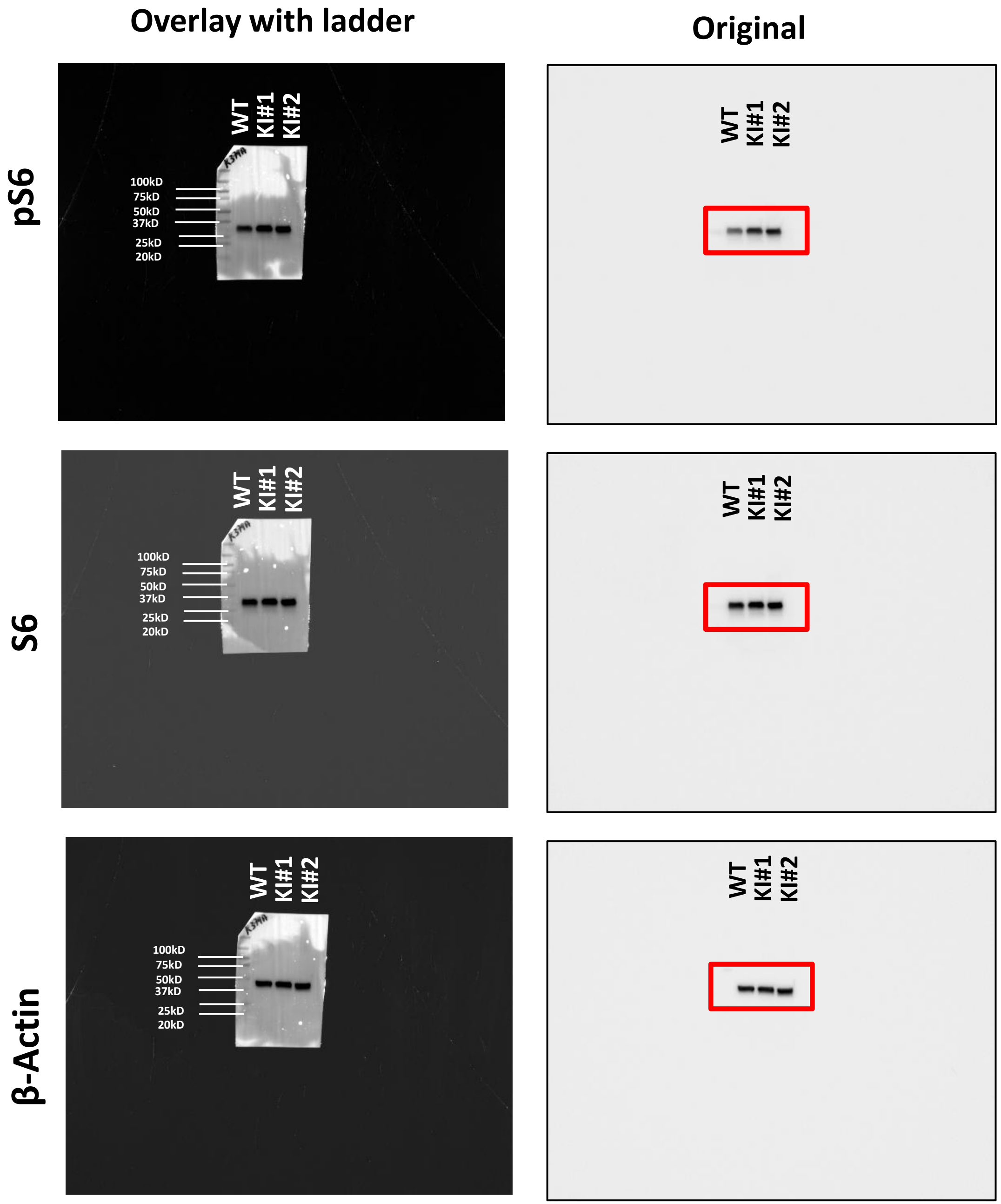

Area used for assembled the figure

pS6
Predicted mol. Wt: 32kDA
Observed mol. Wt: 32kDA
S6
Predicted mol. Wt: 32kDA
Observed mol. Wt: 32kDA

Blot: K379A

Unedited blots associated with Figure 3C_UM-UC-3_KI#1_Top left

Overlay with ladder | **Original**

GLS1

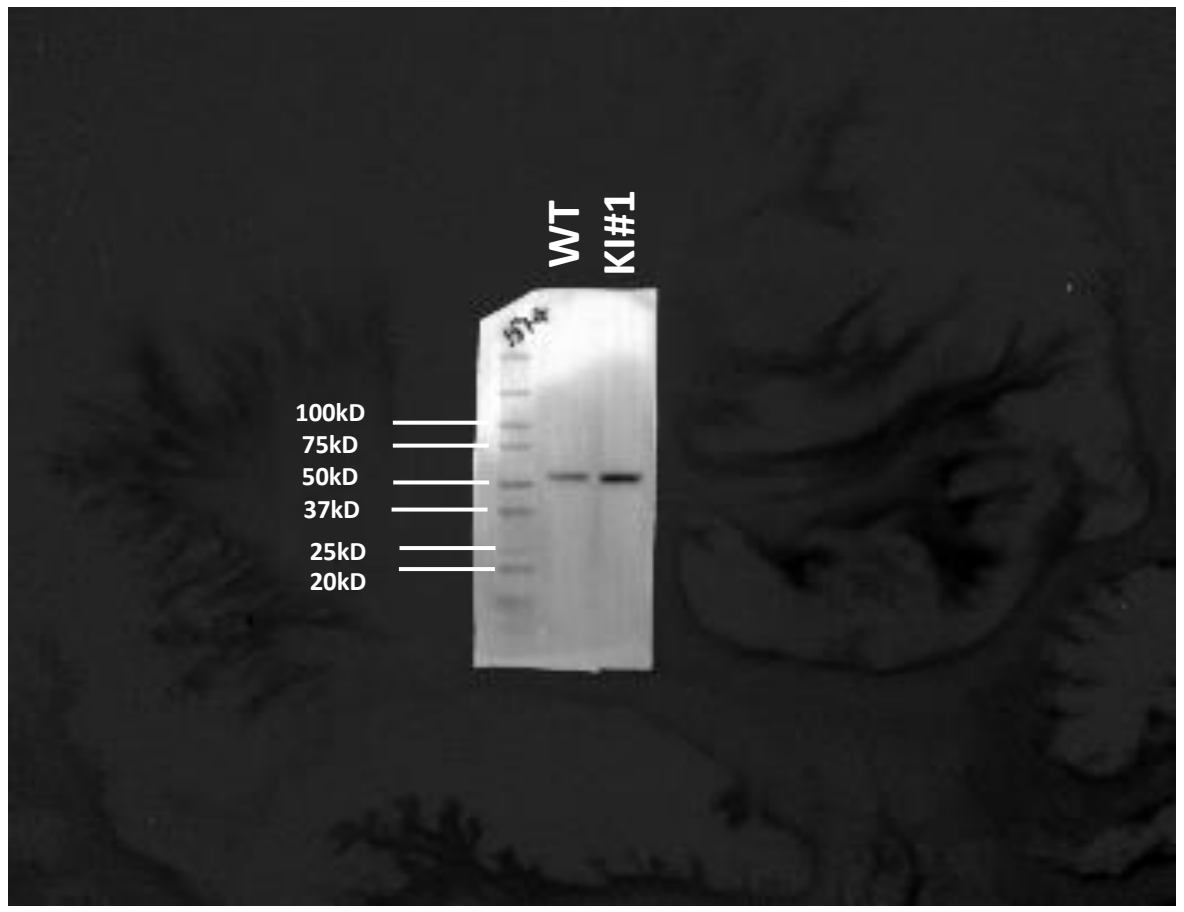

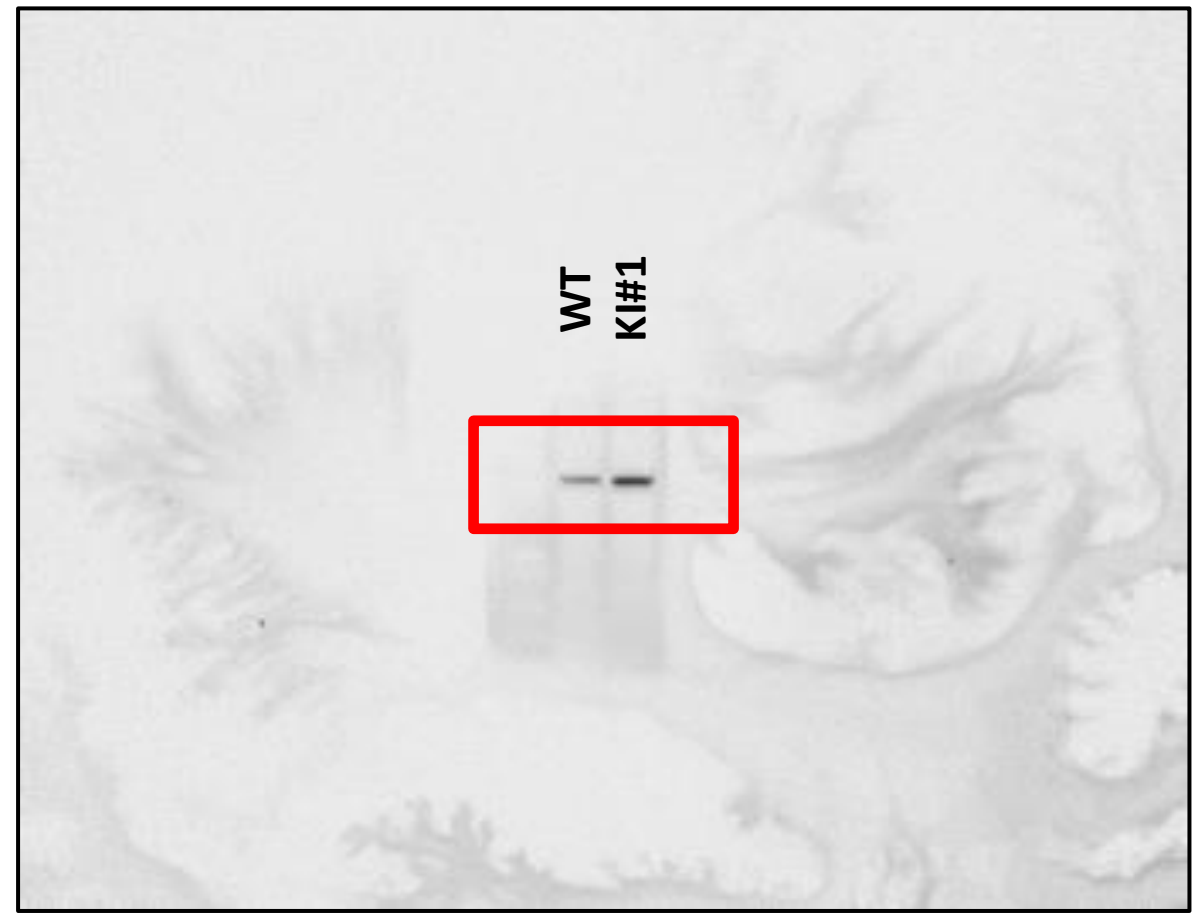

GAPDH

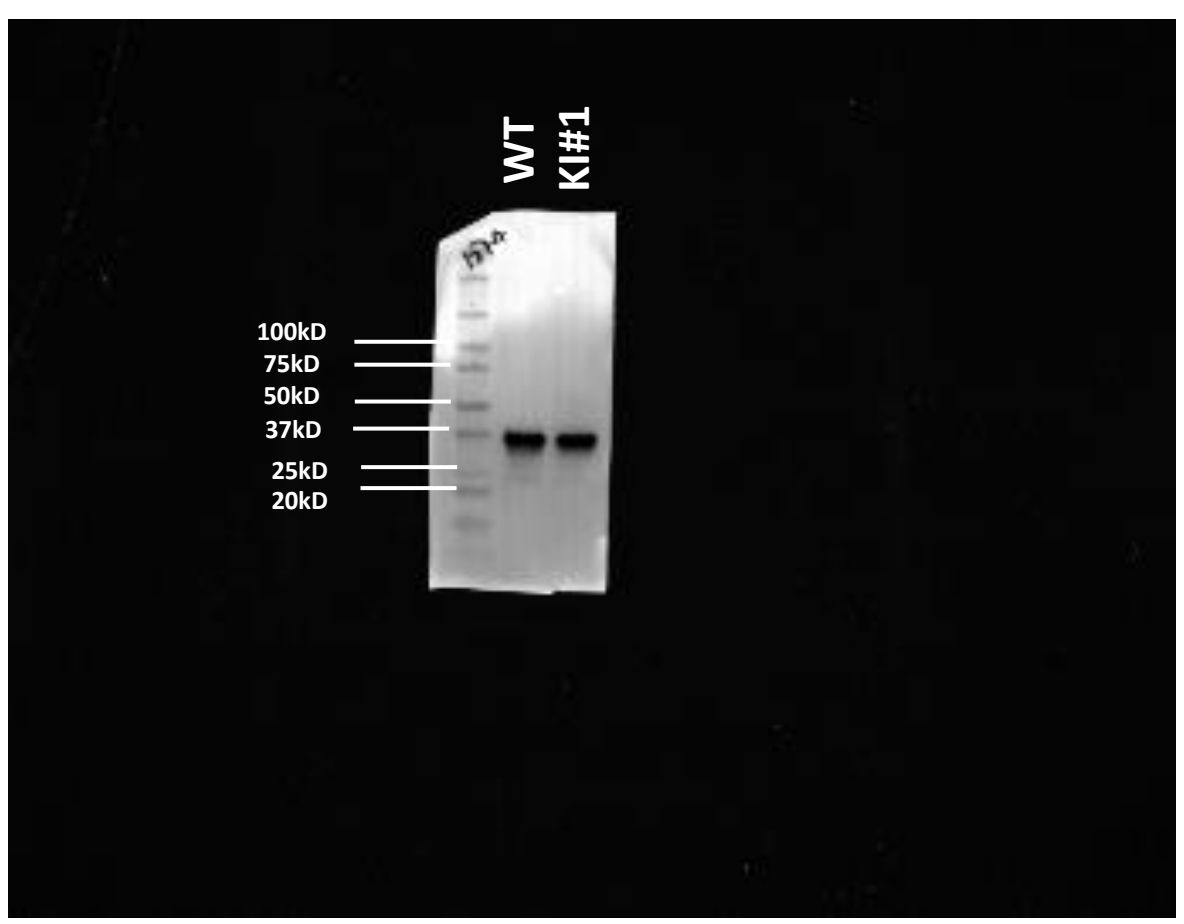

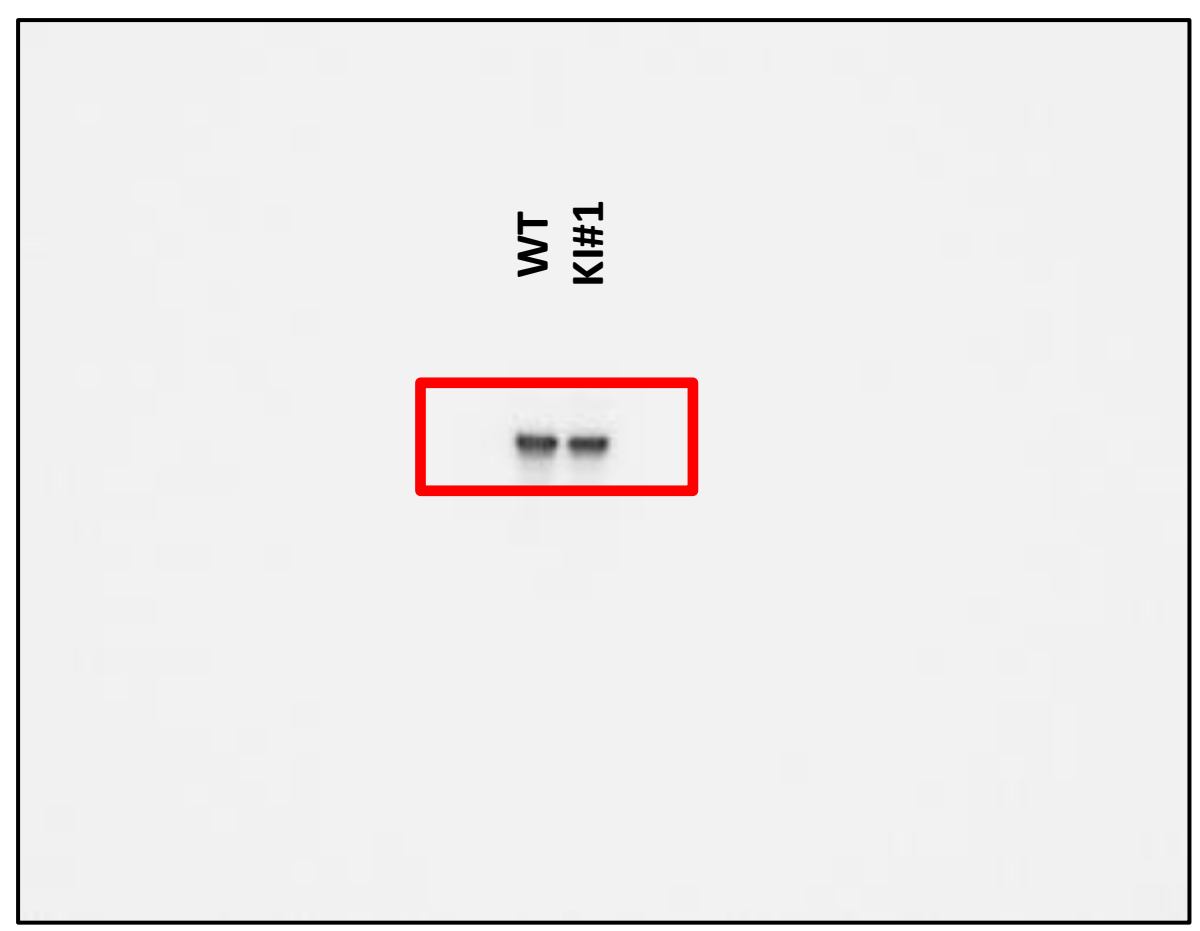

Area used for assembled the figure

GLS1
Predicted mol. Wt: 55-65kDA
Observed mol. Wt: 60kDA

Blot:157a

Unedited blots associated with Figure 3C _UM-UC-3_KI#2_Top right

Overlay with ladder

Original

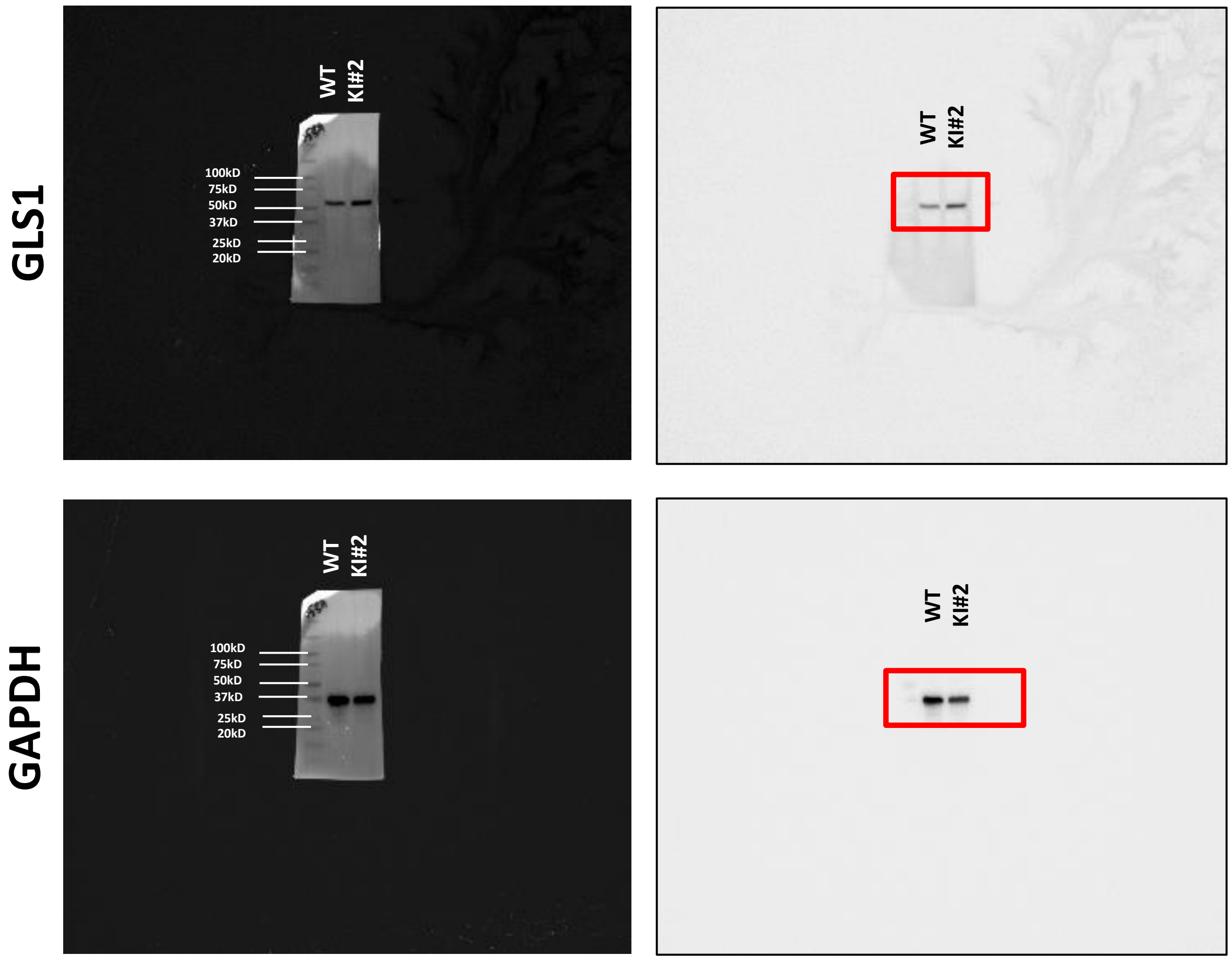

Area used for assembled the figure

GLS1
Predicted mol. Wt: 55-65kDA
Observed mol. Wt: 60kDA

Blot:158a

Unedited blots associated with Figure 3C _UM-UC-3_KI#1_Bottom left

Overlay with ladder **Original**

FASN

GAPDH

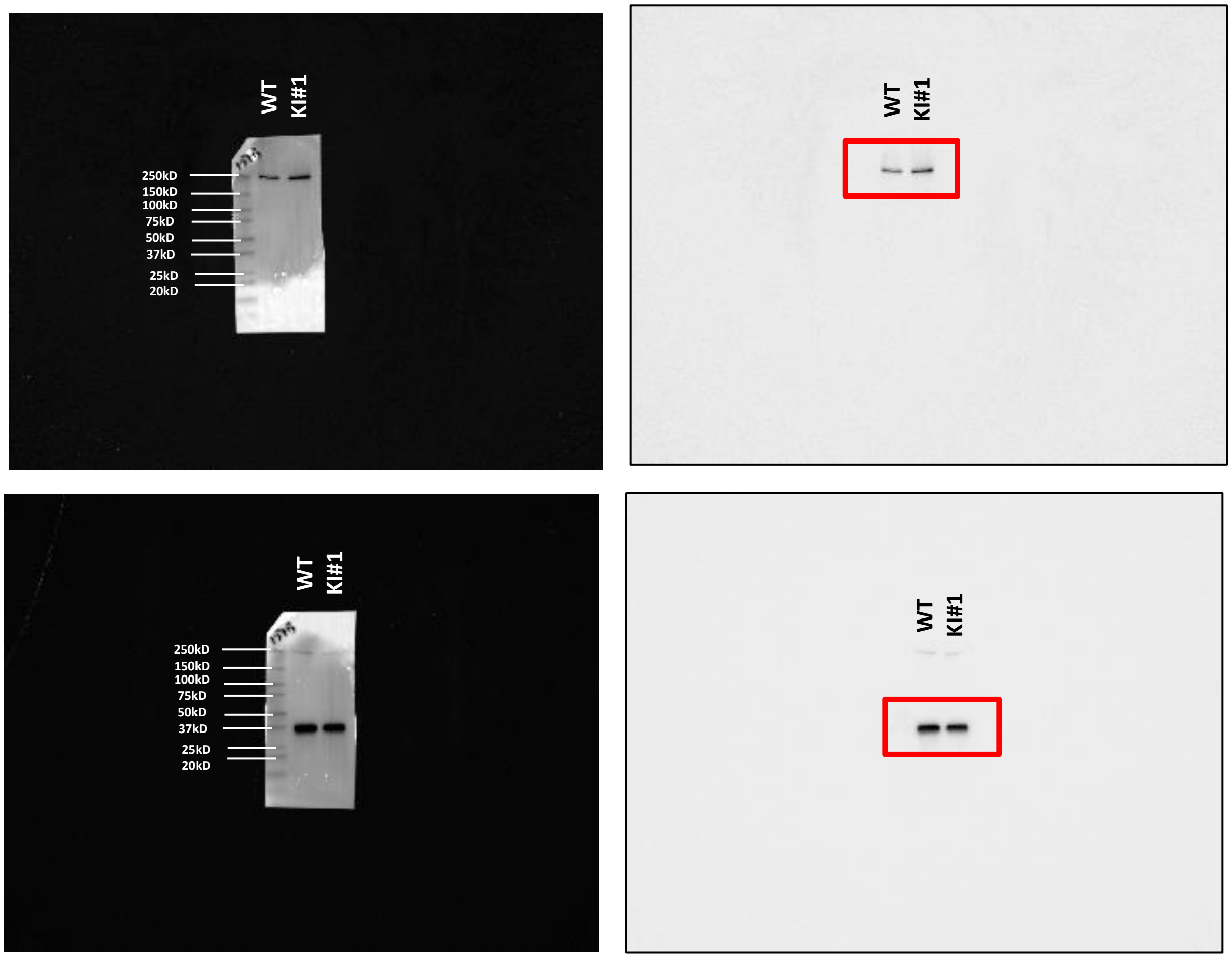

Area used for assembled the figure

FASN
Predicted mol. Wt: 273kDA
Observed mol. Wt: 250kDA

Blot:157b

Unedited blots associated with Figure 3C _UM-UC-3_KI#2_Bottom right

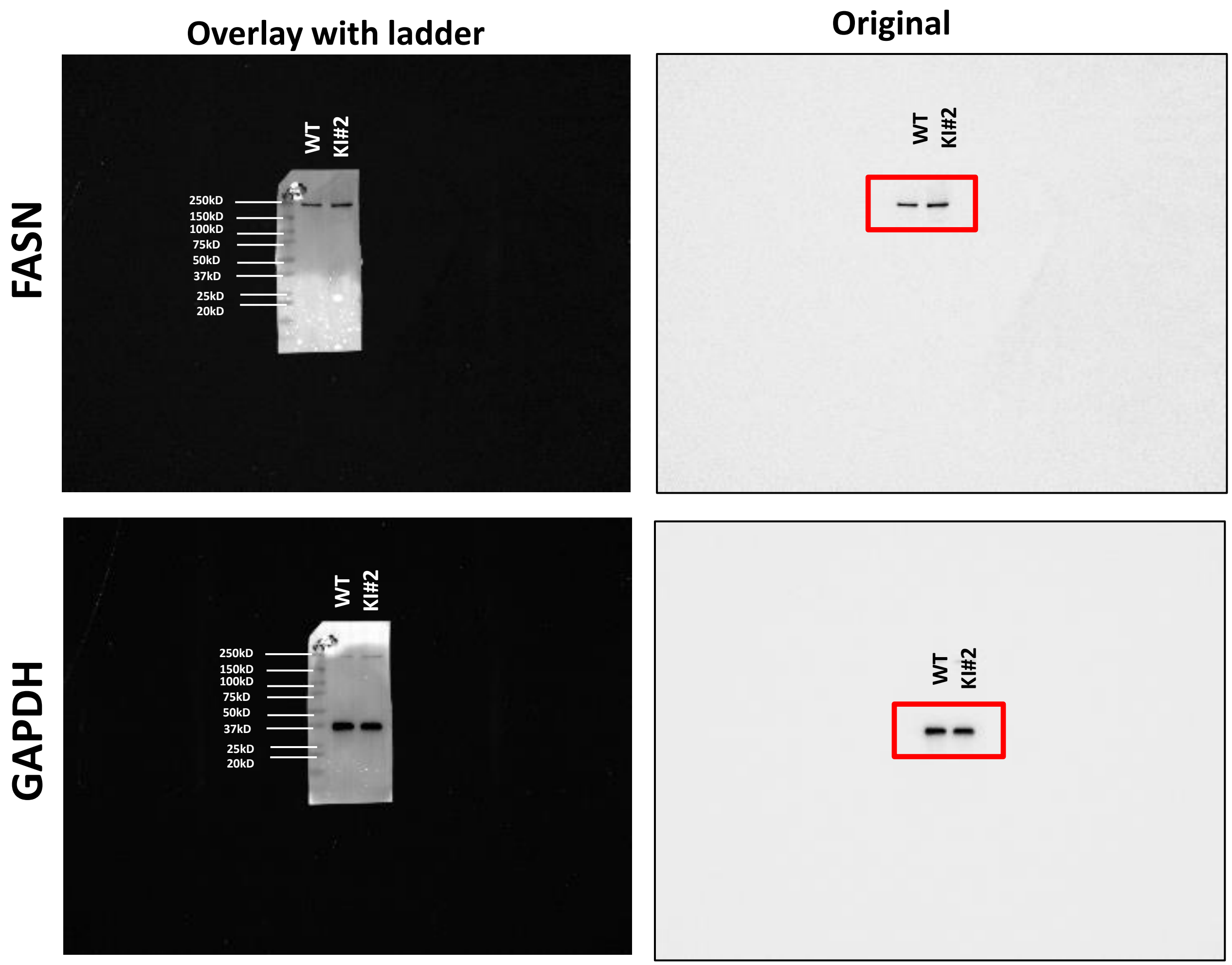

Area used for assembled the figure

FASN
Predicted mol. Wt: 273kDA
Observed mol. Wt: 250kDA

Blot:158 b

Unedited blots associated with Figure 3D_SCaBER_KI#1_Top left

Overlay with ladder | Original

GLS1

GAPDH

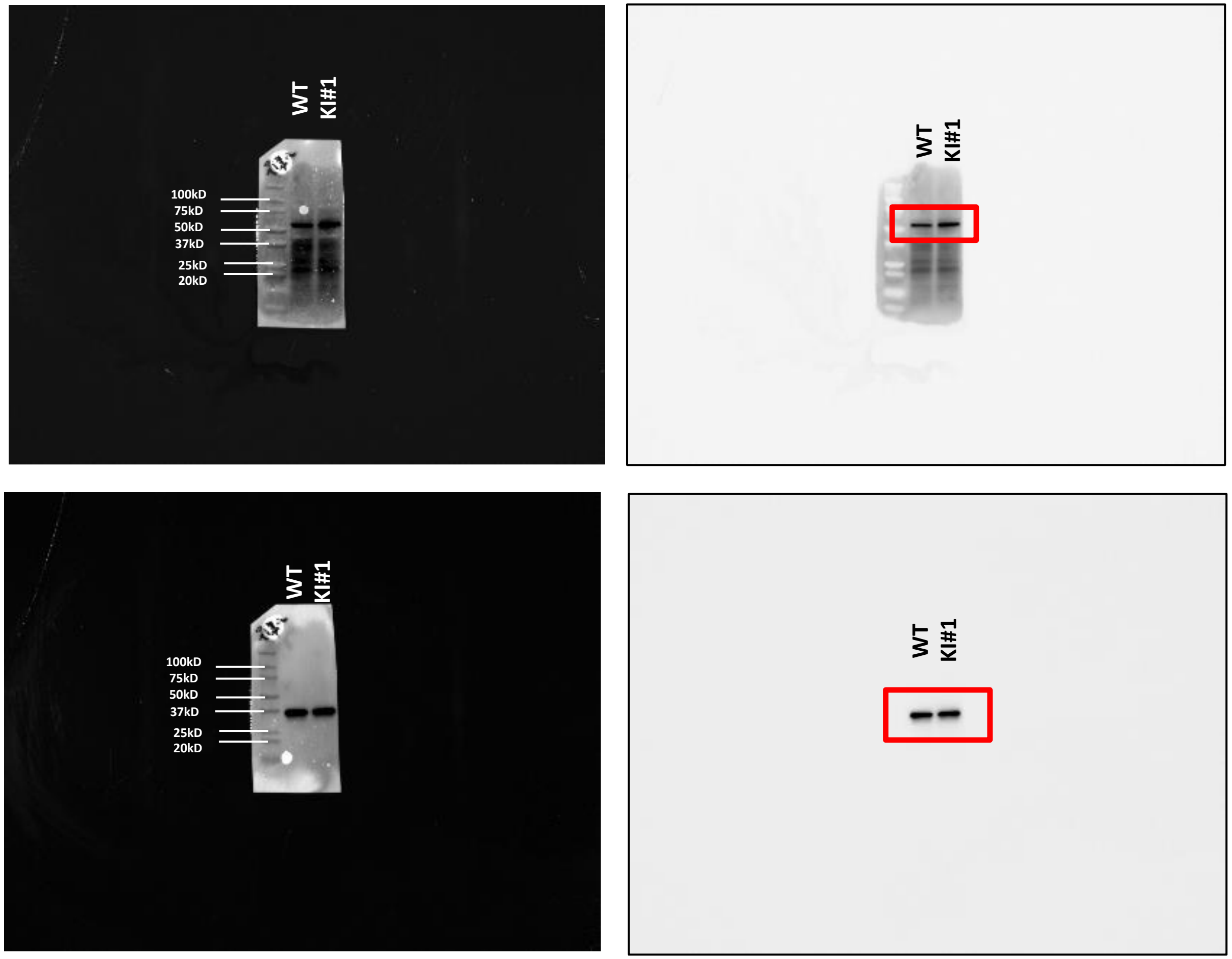

Area used for assembled the figure

GLS1
Predicted mol. Wt: 55-65kDA
Observed mol. Wt: 60kDA

Blot:217 a

Unedited blots associated with Figure 3D_SCaBER_KI#2_Top right

Overlay with ladder

Original

GLS1

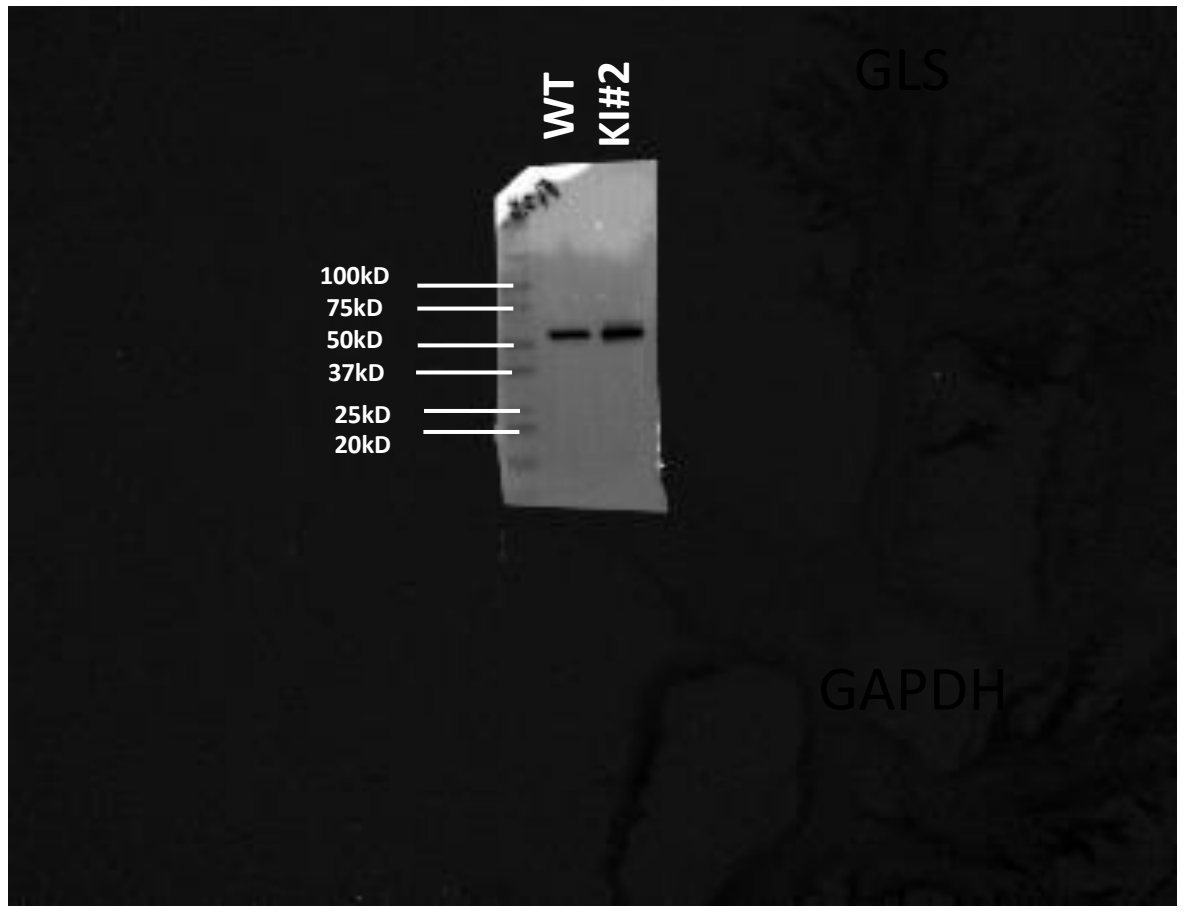

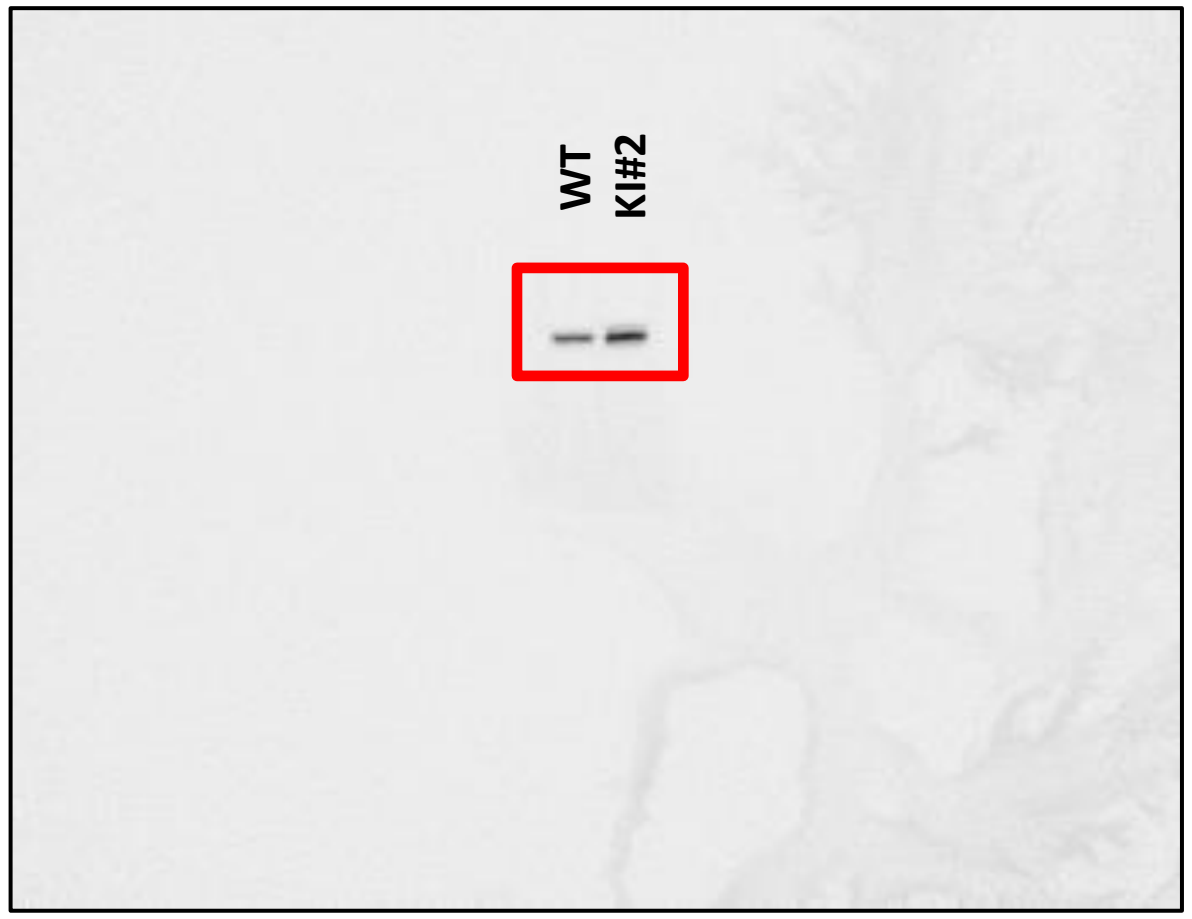

GAPDH

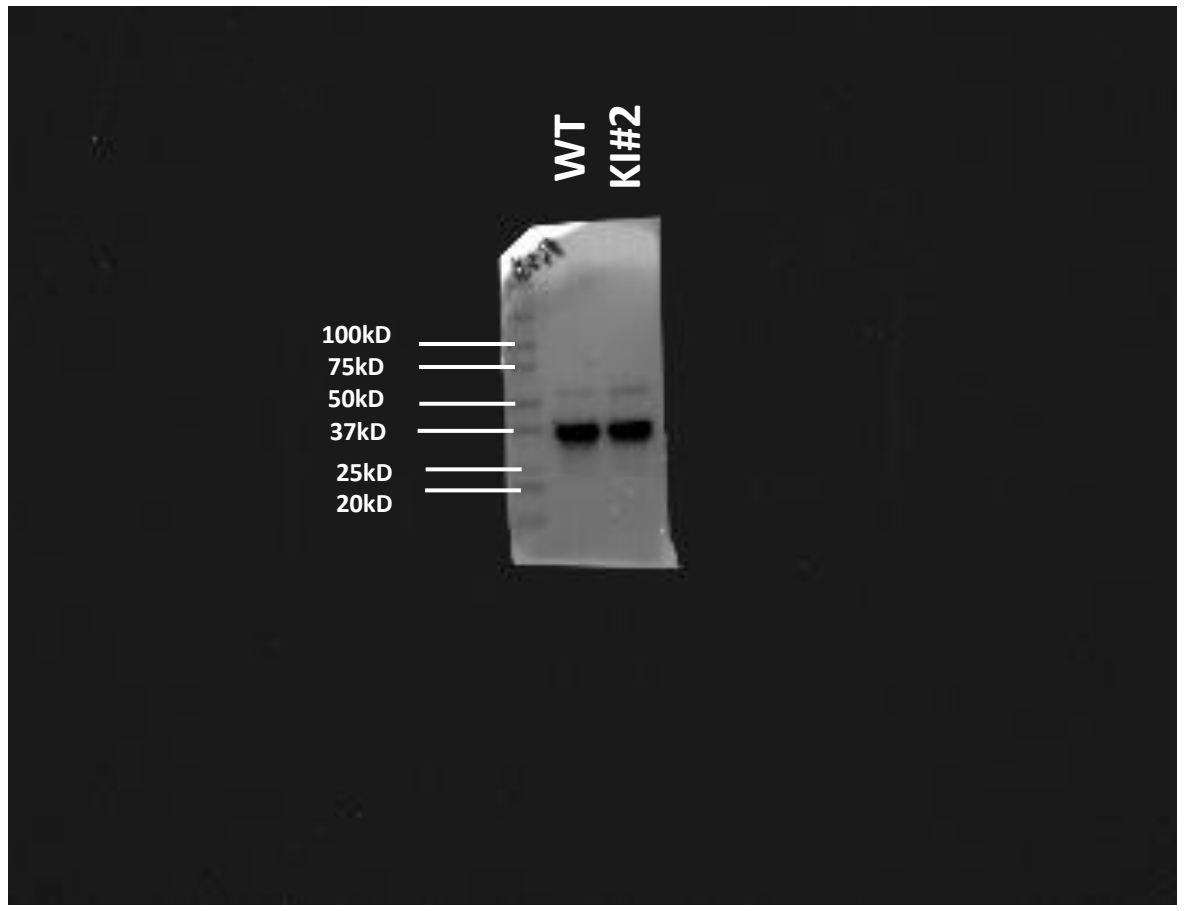

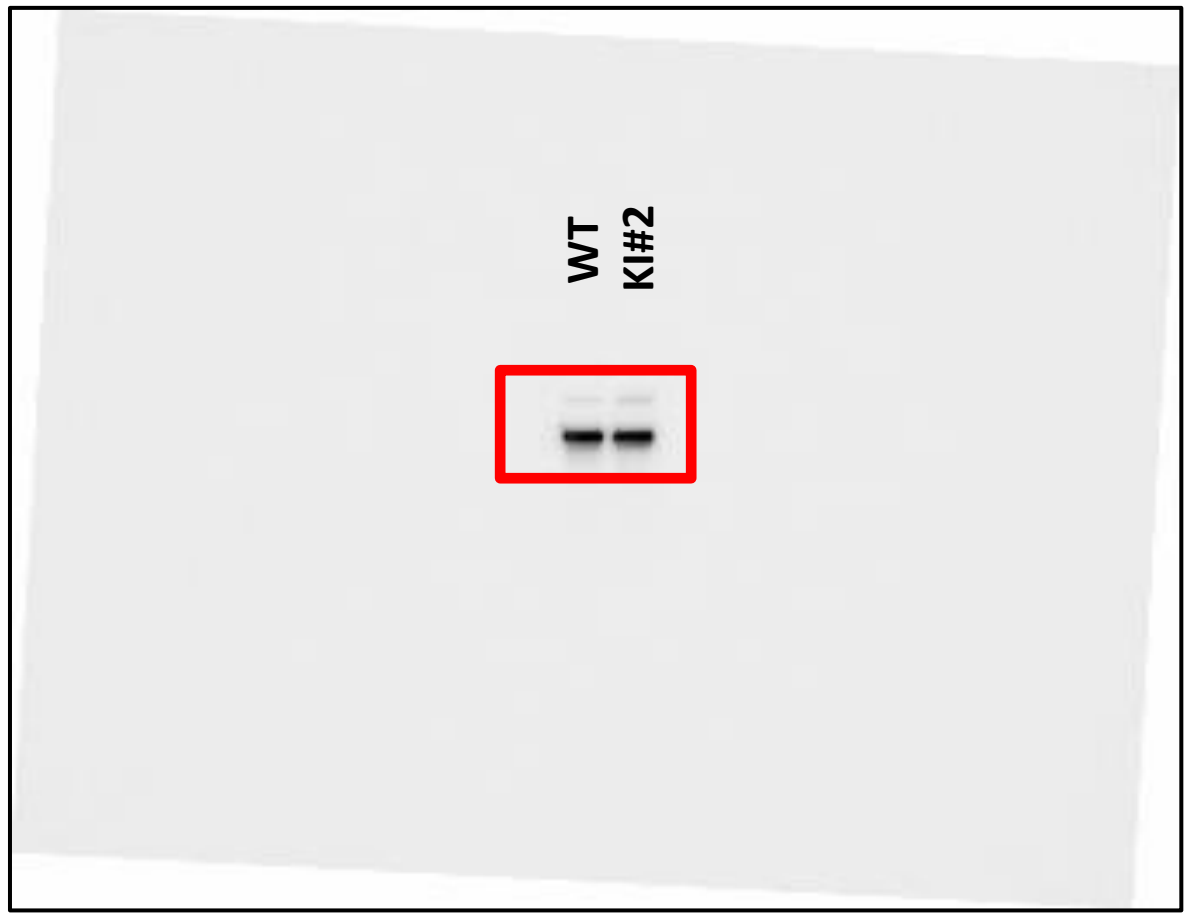

Area used for assembled the figure

GLS1
Predicted mol. Wt: 55-65kDA
Observed mol. Wt: 60kDA

Blot:160a

Unedited blots associated with Figure 3D_SCaBER_KI#1_Bottom left

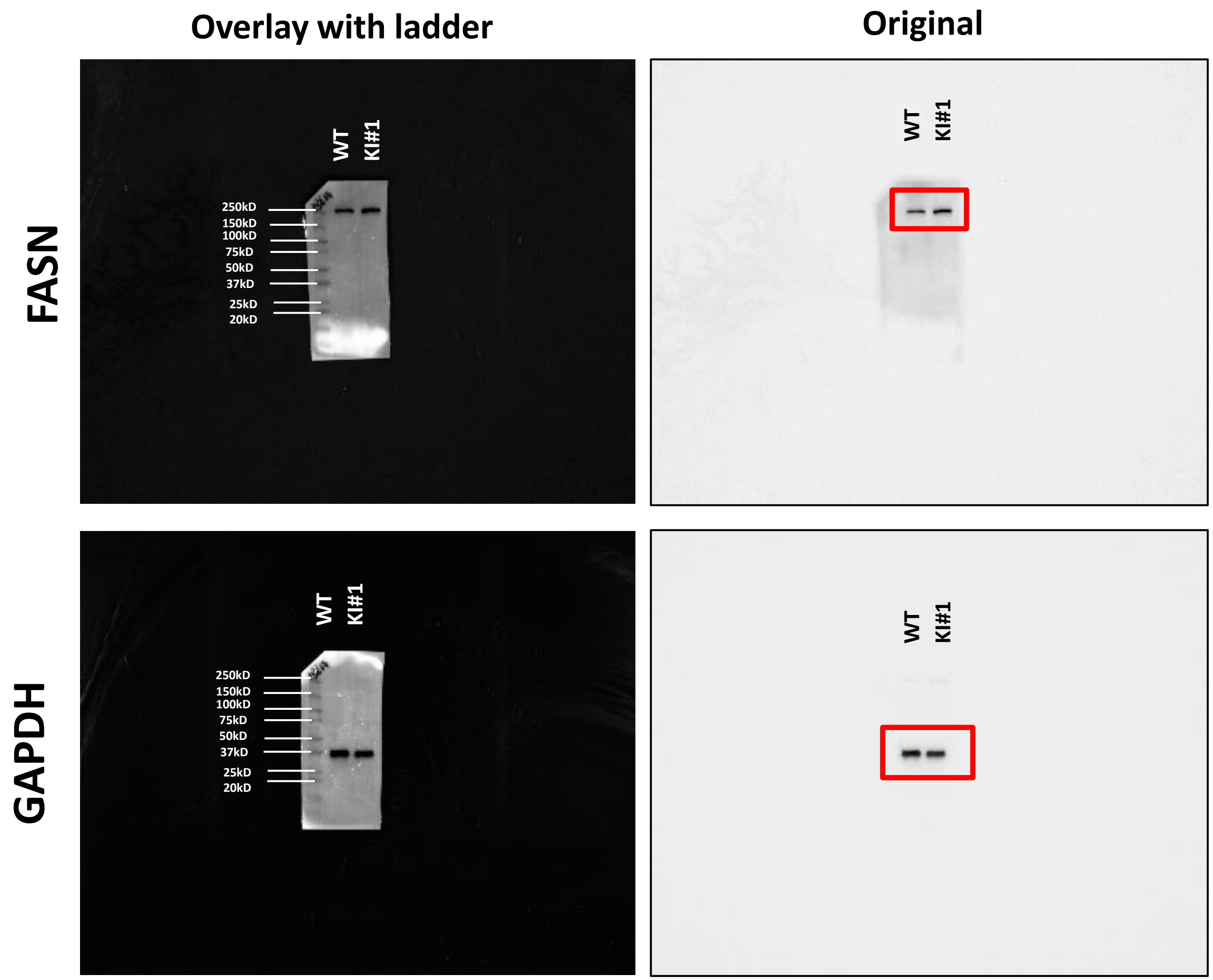

Area used for assembled the figure

FASN: 1 in 1000, CST
GAPDH: 1 in 5000, CST

FASN
Predicted mol. Wt: 273kDA
Observed mol. Wt: 250kDA

Blot:186A

Unedited blots associated with Figure 3D_SCaBER_KI#2_Bottom right

Overlay with ladder | **Original**

FASN

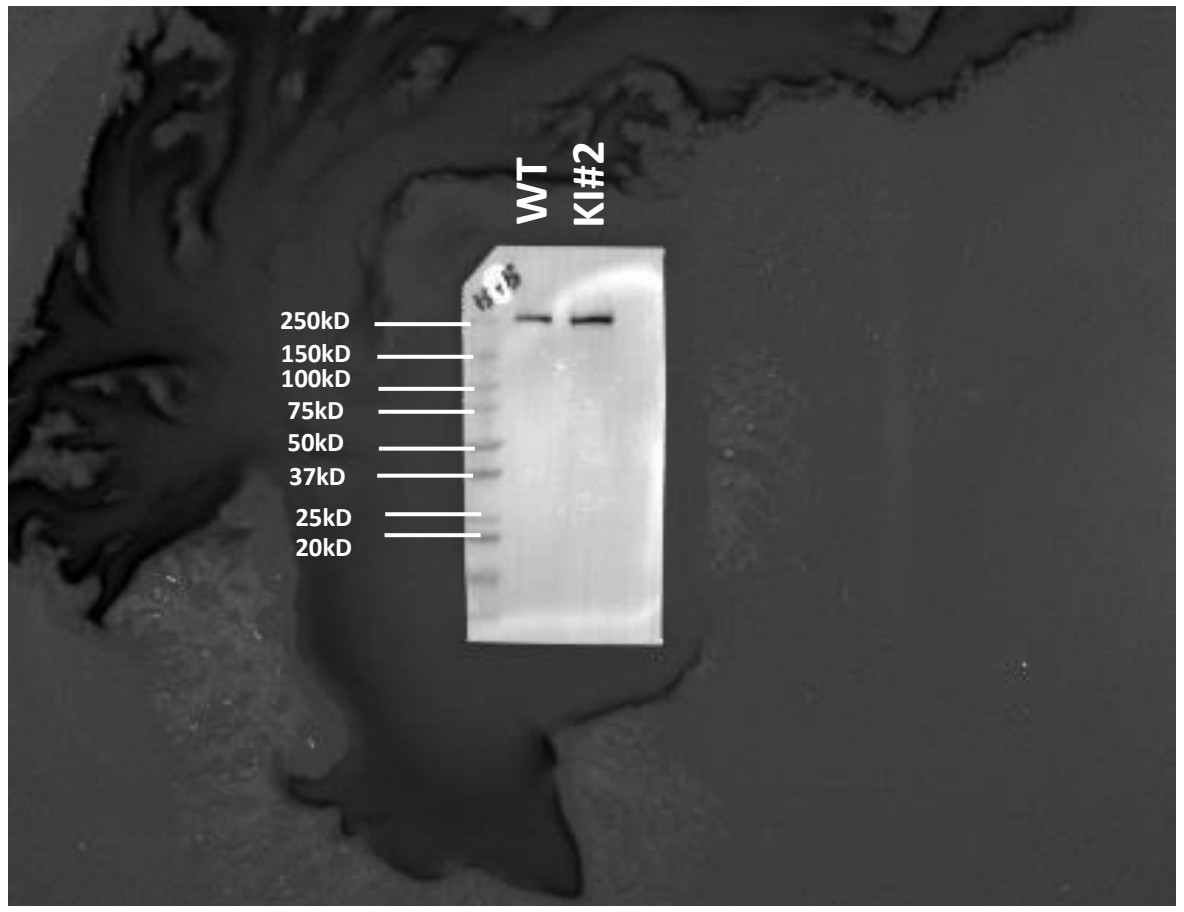

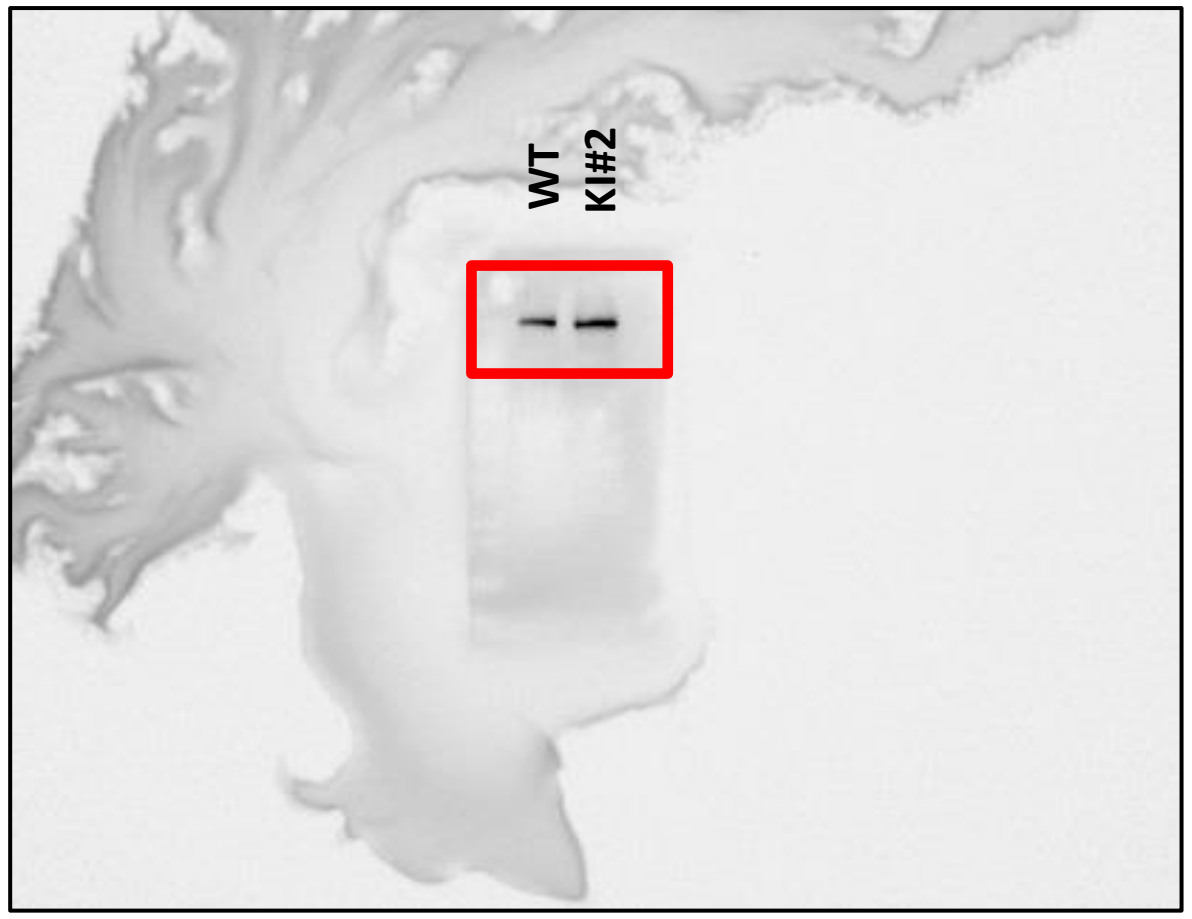

GAPDH

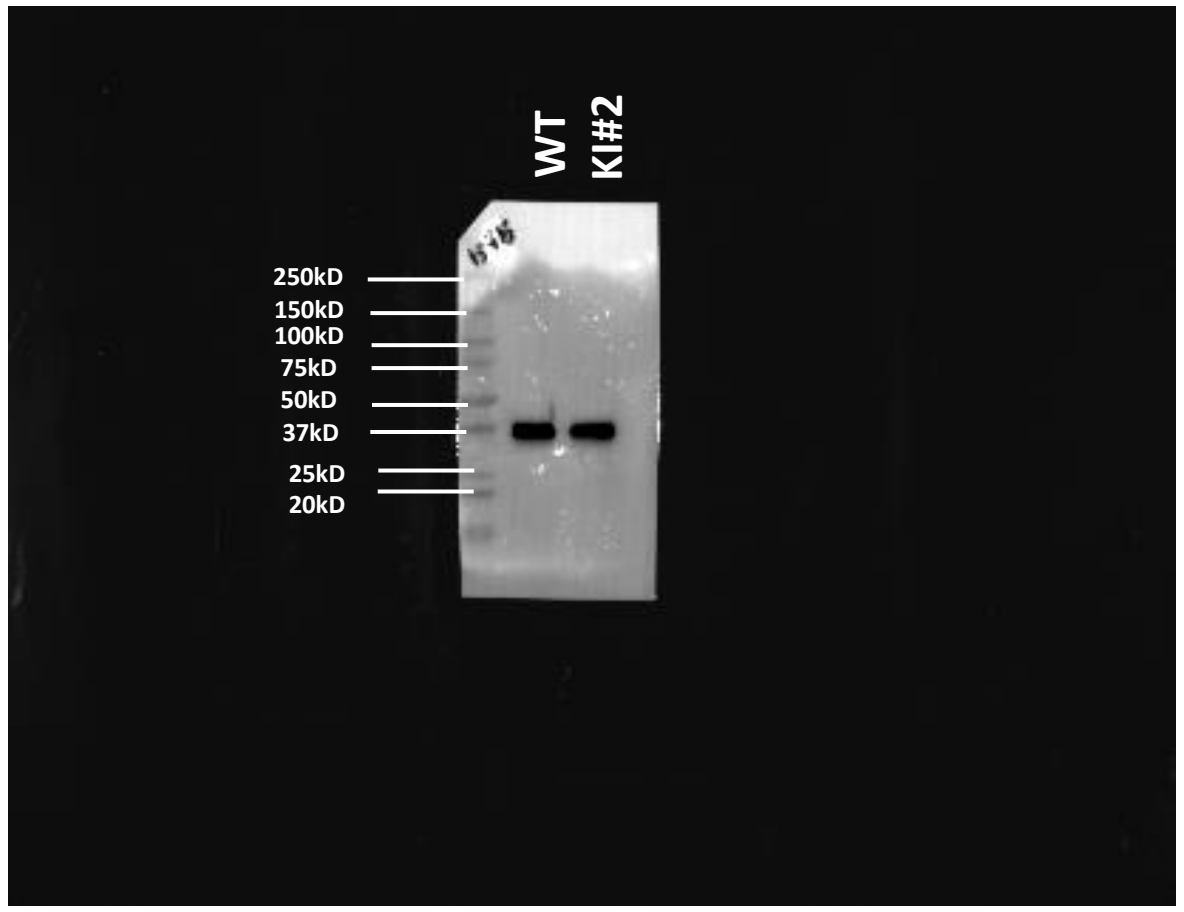

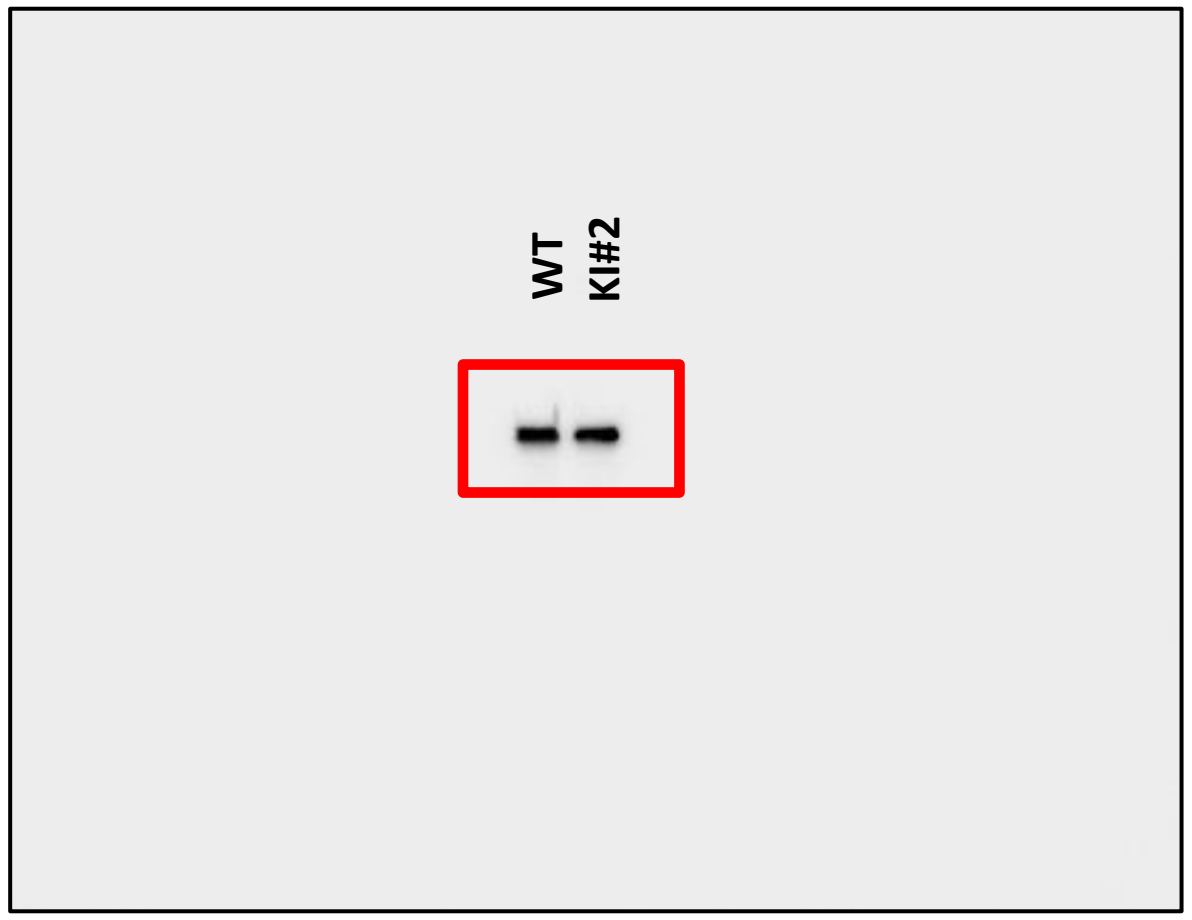

Area used for assembled the figure

FASN
Predicted mol. Wt: 273kDA
Observed mol. Wt: 250kDA

Blot:187 B

Raw images associated with Figure 5B

SCaBER WT

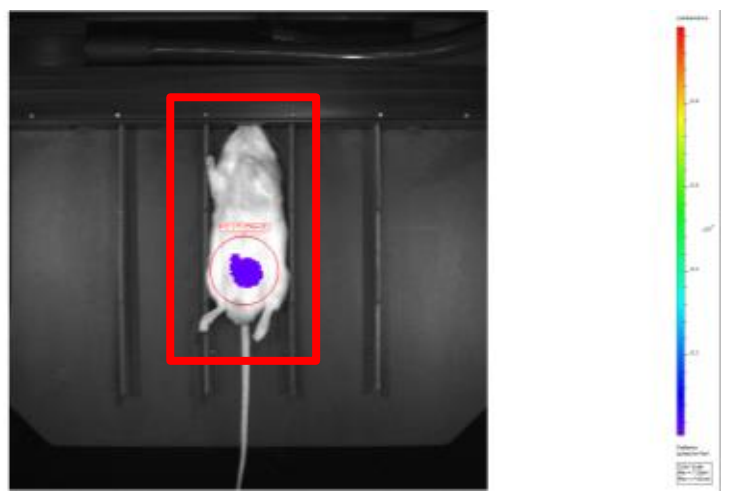

SCaBER PIK3CA^{mut} KI#1

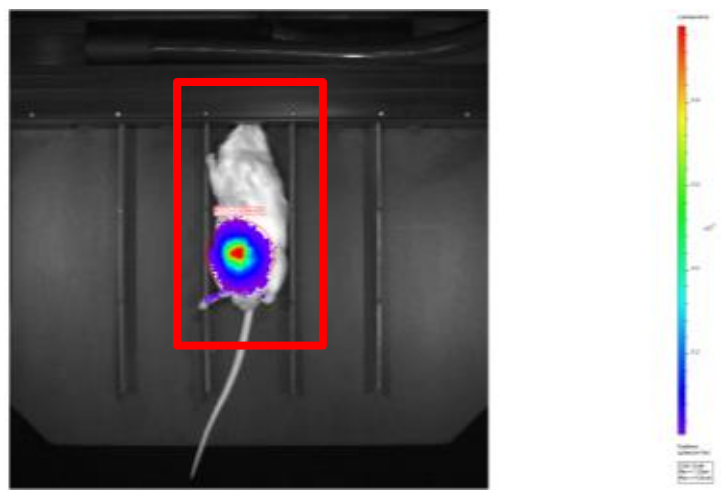

SCaBER PIK3CA^{mut} KI#2

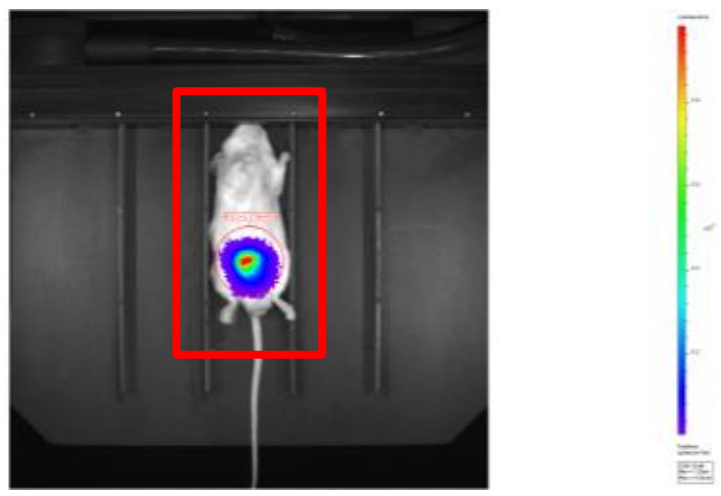

Area used for assembled the figure

Unedited blots associated with Supplementary Figure 1B_UM-UC-3_ top panel

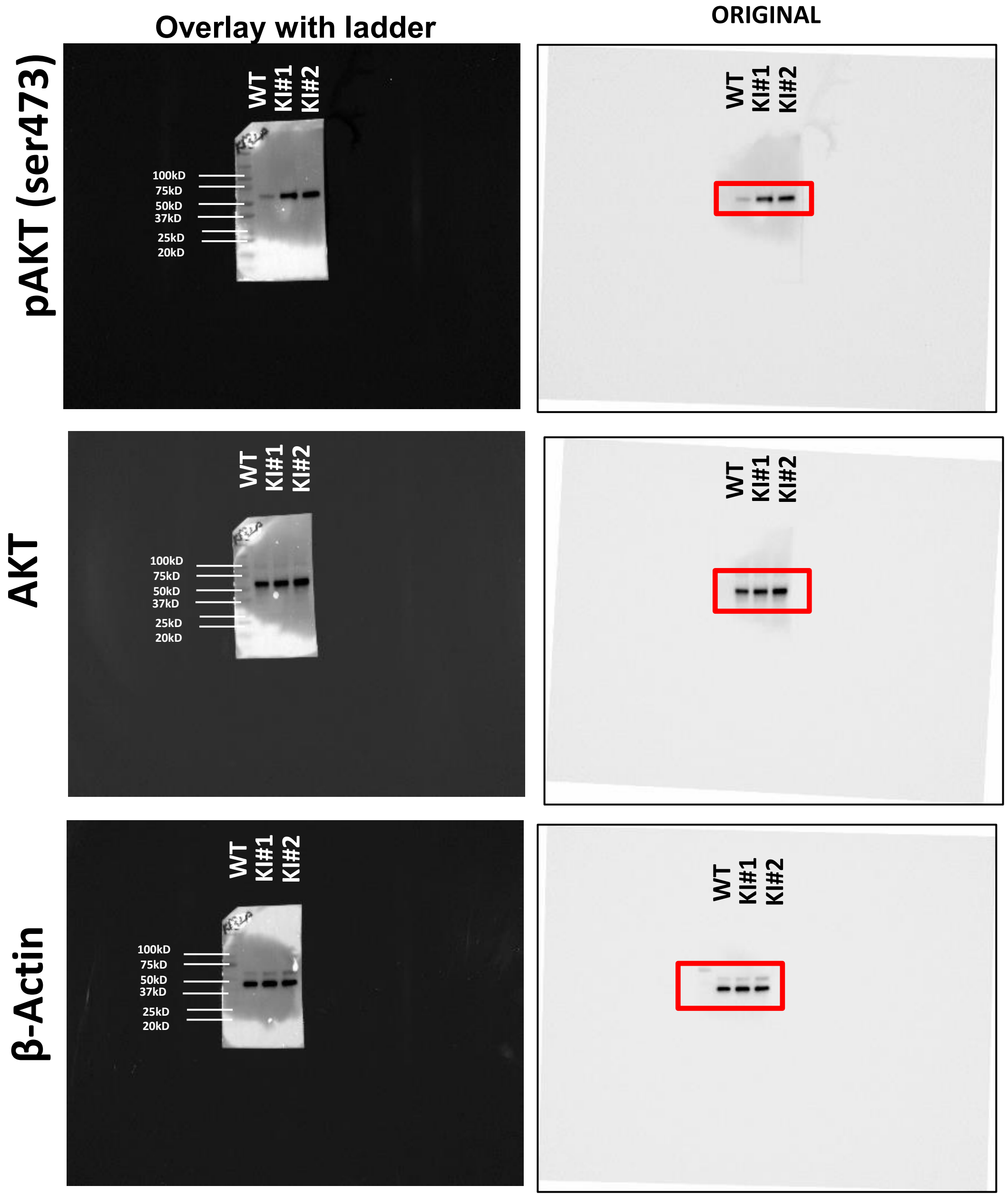

Area used for assembled the figure

pAKT ser473
Predicted mol. Wt: 60kDA
Observed mol. Wt: 60kDA
AKT
Predicted mol. Wt: 60kDA
Observed mol. Wt: 60kDA

Blot: K632A

Unedited blots associated with Supplementary Figure 1B_UM-UC-3_ bottom panel

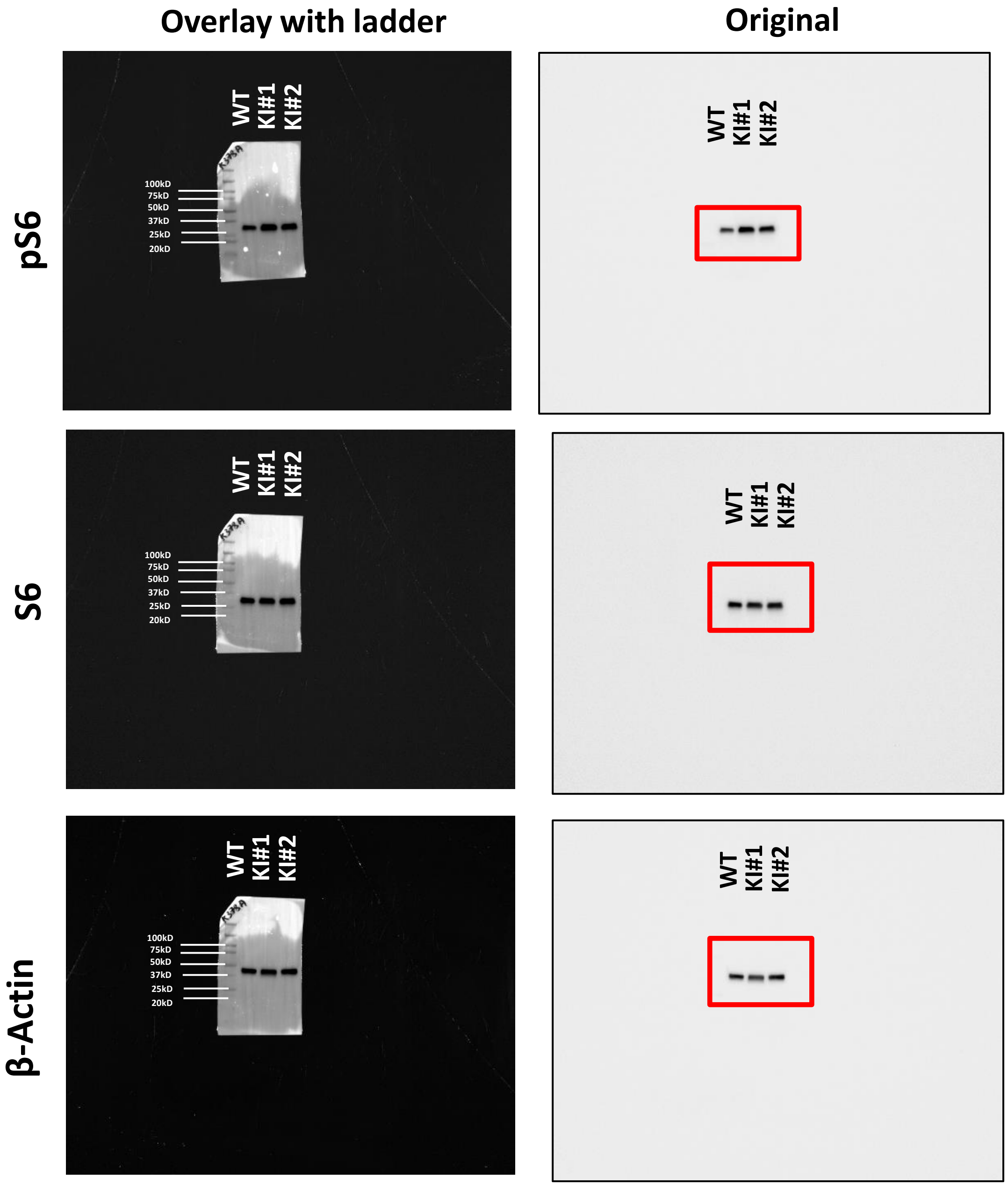

Area used for assembled the figure

pS6
Predicted mol. Wt: 32kDA
Observed mol. Wt: 32kDA
S6
Predicted mol. Wt: 32kDA
Observed mol. Wt: 32kDA

Blot: K378A
